# Extraction of Omega-3 Fatty Acids from Atlantic Sea Cucumber (*Cucumaria frondosa*) Viscera Using Supercritical Carbon Dioxide

**DOI:** 10.3390/md22080366

**Published:** 2024-08-12

**Authors:** Jianan Lin, Guangling Jiao, Marianne Su-Ling Brooks, Suzanne M. Budge, Azadeh Kermanshahi-pour

**Affiliations:** 1 Biorefining and Remediation Laboratory, Department of Process Engineering and Applied Science, Dalhousie University, Halifax, NS B3J 1B6, Canada; jianan.lin@dal.ca; 2 AKSO Marine Biotech Inc., Hacketts Cove, NS B3Z 3K7, Canada; 3 Department of Process Engineering and Applied Science, Dalhousie University, Halifax, NS B3H 4R2, Canada

**Keywords:** *Cucumaria frondosa*, supercritical carbon dioxide, supercritical fluid extraction, sea cucumber by-products, omega-3 fatty acids, ethanol-soaking pre-treatment, green extraction

## Abstract

This study explores the potential of *Cucumaria frondosa* (*C. frondosa*) viscera as a natural source of omega-3 FAs using supercritical carbon dioxide (scCO_2_) extraction. The extraction conditions were optimized using a response surface design, and the optimal parameters were identified as 75 °C and 45 MPa, with a 20 min static and a 30 min dynamic extraction, and a 2:1 ethanol to feedstock mass ratio. Under these conditions, the scCO_2_ extraction yielded higher FAs than the solvent-based Bligh and Dyer method. The comparative analysis demonstrated that scCO_2_ extraction (16.30 g of FAs/100 g of dried samples) yielded more fatty acids than the conventional Bligh and Dyer method (9.02 g, or 13.59 g of FAs/100 g of dried samples with ultrasonic assistance), indicating that scCO_2_ extraction is a viable, green alternative to traditional solvent-based techniques for recovering fatty acids. The pre-treatment effects, including drying methods and ethanol-soaking, were investigated. Freeze-drying significantly enhanced FA yields to almost 100% recovery, while ethanol-soaked viscera tripled the FA yields compared to fresh samples, achieving similar EPA and DHA levels to hot-air-dried samples. These findings highlight the potential of sea cucumber viscera as an efficient source of omega-3 FA extraction and offer an alternative to traditional extraction procedures.

## 1. Introduction

Sea cucumbers, marine invertebrates under the phylum of Echinoderms and the class Holothuroidea, are ideal functional foods with high nutritional value, which have been exploited and used for hundreds of years as food delicacies and medicines for a wide variety of diseases, such as diabetes [1,2,3]. Several studies have proven that sea cucumbers are rich sources of essential amino acids, proteins, vitamins (vitamin A, B1, B2, and B3), mineral elements (zinc, iron, magnesium, calcium), and various bioactive molecules (e.g., FAs, saponins, sulfated polysaccharides, and collagens) with diverse therapeutic properties, such as anticancer, anti-inflammatory, anticoagulant, etc. [2,4]. Therefore, sea cucumbers have attracted increasing interest in developing techniques to isolate valuable compounds and understanding their potential as pharmaceuticals, nutraceuticals, and functional food.

**Figure 1:**
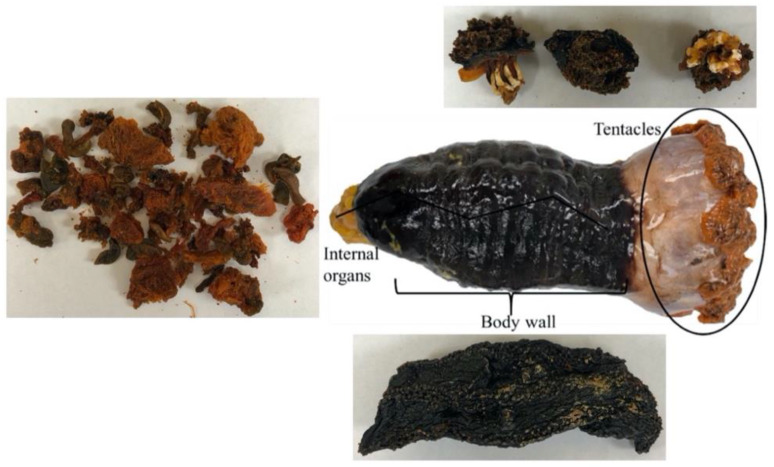
The structure of sea cucumber, *C. frondosa* (fresh sea cucumber entirety and dehydrated parts). Adapted from Hossain et al. (2020) [3]; the pictures of dehydrated parts were taken on samples provided by AKSO Marine Biotech Inc. (Hacketts Cove, NS, Canada).

*C. frondosa* (Figure 1), also known as the orange-footed sea cucumber or the northern sea cucumber, is a dendrochirotic sea cucumber species with equally developed dendritic tentacles [5]. It comprises three parts, including body walls, tentacles, and viscera [3]. The general practice of preparing sea cucumbers for the food market relies on the removal of tentacles and internal organs during processing; thus, a considerable amount of sea cucumber by-products containing valuable matter end up in landfills. It was reported that discarded viscera represented up to 50% of the Atlantic sea cucumber biomass [6]. Currently, sea cucumbers are mainly sold as frozen and dried sea cucumber body walls. However, all the constituents are edible, and by-products (i.e., viscera and tentacles) contain various bioactive compounds. For example, Zhong and his coworkers found sea cucumbers with viscera had higher antioxidant activity due to the astaxanthin-rich orange viscera [7]. The growing market for nutraceutical supplements provides an opportunity to enhance the profit margins of sea cucumbers by generating value from by-products that are typically discarded as waste. Adverse environmental impacts of wastes generated from seafood processing have driven research into the extraction of value-added compounds, such as lipids and astaxanthin in shrimp residues [8,9,10], astaxanthin in blue crab shell wastes [11], and lipids in fish by-products [12,13]. However, there is limited research into the utilization of sea cucumber by-products.

The market demand for foods or relevant nutraceuticals from natural sources that are high in PUFAs, particularly omega-3 FAs, including EPA and DHA, has led to an increase in sea cucumber consumption in recent years [14]. EPA and DHA are long-chain omega-3 PUFAs with more than 20 carbons. They promote anti-inflammatory activities and support cardiovascular health, brain function, metabolism and immune function, and anticancer [15]. Recent studies highlight the potential of *C. frondosa* viscera from Atlantic Canada as a rich source of lipids, particularly PUFAs and EPA, making them ideal candidates for omega-3-enriched product development. Researchers found lipid contents ranging from 0.7 to 4.68% of wet biomass or 20.81 to 28.87% on a dry weight basis, with EPA making up a significant portion of the fatty acids (24–43% in the total FAs) [7,16,17]. Further research on dried *C. frondosa* viscera confirms their high lipid (23–26% on a dry weight basis), PUFA (31–32% in the total FAs), and EPA (28–29% in the total FAs) content, reinforcing their value for omega-3 extraction [16]. These levels are considerably higher than those found in *Apostichopus japonicus* [18].

To the best of our knowledge, current studies on sea cucumbers mainly used conventional methods involving organic solvents such as chloroform and n-hexane for lipid extraction [7,17,19]. However, chlorinated solvents such as chloroform are associated with health and environmental issues, whereas other solvents such as n-hexane and n-heptane demonstrate low extraction yield [8]. The cost of solvents, their health and environmental concerns, and strict requirements for product purity challenge the use of organic solvents to extract natural products at industrial levels. Thus, to eliminate/reduce the use of organic solvents, researchers are motivated to seek greener alternatives to conventional extraction methods. There has been a growing trend toward the scCO_2_ extraction method in recent decades.

ScCO_2_ is CO_2_ in a supercritical fluid state, where it is maintained at or above its critical temperature of approximately 31 °C and critical pressure of around 7.38 MPa [20]. It is generally recognized as safe, non-toxic, non-flammable, and inexpensive. Additionally, CO_2_ is recyclable and can be easily removed from products due to its high volatility at ambient temperature and pressure [8,21]. Compared with traditional processes, scCO_2_ extraction shows a high selectivity for bioactive compounds and avoids the use of toxic organic solvents; meanwhile, due to the characteristics of supercritical fluids—low viscosity and high diffusivity—this technique improves the extraction efficiency by enhancing mass transfer [21]. Research reveals that scCO_2_ extraction is superior to conventional methods for isolating FAs and omega-3 FAs from various marine sources, including *Sargassum hemiphyllum* macroalgae [22], *Pavlova* sp. microalgae [23], and northern shrimp [10], with FAs and lipid yields often exceeding those achieved with solvent extraction. Nevertheless, the most significant limitation associated with scCO_2_ extraction is that this technique is not well suited for extracting polar compounds, but adding co-solvents can modify the non-polar nature of scCO_2,_ enhancing extraction efficiency [24]. In light of current research, few published scientific papers employed scCO_2_ extraction to isolate lipids from sea cucumbers and their by-products; only four patents in China briefly reported the application of this technique for oil extraction from sea cucumbers [25,26,27,28].

Even though scCO_2_ extraction has been extensively studied as a potential technique used in lipid production and has been applied to extract oils from a variety of animal and plant feedstocks, the recovery of omega-3 FAs from *C. frondosa* viscera has not been explored using scCO_2_. Additionally, the impact of pre-treatments (e.g., drying methods) and the use of co-solvent ethanol on extraction has only been explored to a limited extent [9,29]. Thus, this study aims to investigate the effects of process variables, including temperature, pressure, extraction time, co-solvent-to-feedstock ratio, drying methods, and ethanol-soaking pre-treatment, on the lipid extraction yields from *C. frondosa* viscera. The condition with the highest extraction yields was found by employing response surface design to optimize the extraction process. It is hypothesized that scCO_2_ extraction coupled with pre-treatments provides a greener alternative to conventional solvent extractions for obtaining omega-3 fatty acids from *C. frondosa* viscera. Also, this study compares the efficiency of scCO_2_ extraction, the Bligh and Dyer method, and in situ transesterification. In situ transesterification allows the direct conversion of lipids within the biomass into fatty acid methyl esters (FAMEs) without separating lipid extraction and transesterification steps, reducing losses associated with multiple process steps. Thus, the FA contents obtained from in situ transesterification are regarded as a reference to evaluate the extraction performance of scCO_2_ and conventional solvent extraction. The findings are expected to advance the comprehension of scCO_2_ extraction in marine by-product valorization.

## 2. Results and Discussion

### 2.1. ScCO_2_ Extraction of C. frondosa Viscera

#### 2.1.1. Effects of Process Variables on FA Yields

Based on the results obtained from the preliminary screening (in the Supplementary Materials Text S2 and Figures S1–S5), temperature (*TEMP*, 35–75 °C), pressure (*PRESS*, 20–50 MPa), dynamic extraction time (*DET*, 30–70 min), and the mass ratio of co-solvent to feedstock (*MRCSF*, 0-2:1) were used for a response surface design, while static extraction was fixed as 20 min. Table S2 shows the responses studied in scCO_2_ extraction of hot-air-dried *C. frondosa* viscera on the basis of the central composite inscribed (CCI) design. The response surface methodology (RSM) provided information about the importance and significance of individual factors and was hence used to determine the effects of process variables on extraction yields.

The full quadratic regression models in uncoded levels for yields of FAs and the selected omega-3 FAs are shown in Equations (1) and (2) with significant terms in bold.

FA yields = −3.65 − 0.004 *TEMP* + 0.433 ***PRESS*** + 0.176 *DET* + 7.13 ***MRCSF*** + 0.00028 *TEMP*^2^ − 0.00704 ***PRESS*^2^** − 0.000995 *DET*^2^ − 0.098 *MRCSF*^2^ + 0.00316 *TEMP* × *PRESS* + 0.00002 *TEMP* × *DET −* 0.0721 ***TEMP*** × ***MRCSF*** − 0.00119 *PRESS* × *DET* + 0.0108 *PRESS* × *MRCSF* − 0.0384 *DET* × *MRCSF* (1)

Selected omega-3 yields = −0.44 − 0.0045 *TEMP* + 0.0924 *PRESS* + 0.0392 *DET* + 1.051 *MRCSF* + 0.000182 *TEMP*^2^ − 0.000988 ***PRESS*^2^** − 0.000118 *DET*^2^ − 0.052 *MRCSF*^2^ + 0.000208 *TEMP* × *PRESS −* 0.000131 *TEMP* × *DET* − 0.00838 *TEMP* × *MRCSF* − 0.000525 *PRESS* × *DET* − 0.0015 *PRESS* × *MRCSF* − 0.00338 *DET* × *MRCSF* (2)

The experimental FA yields agreed well with their corresponding predicted values, with an R^2^ of 90.69% (Figure S6a). However, the agreement between predicted and experimental values was relatively poorer for the selected FA yields, with an R^2^ of 72.45% (Figure S6b). That might be caused by inherent characteristics such as low yields of EPA and DHA (~3 g/100 g of dried weight-based samples) introducing error into the data collected. Also, FA yields were obtained through a series of operations, including extraction, transesterification, and GC analysis; as a result, the degradation or loss of FAs during sample work-up would be more likely to introduce error into the yields of the selected omega-3 FA yields than total FA yields. However, an R^2^ of 72.45% suggests that 72.45% of the variance in the dependent variable can be explained by the independent variables included in the model, which is generally considered a good level of explanation, indicating a strong model. The results in Table S3 exhibit that the lack of fit of these two models was not significant (P at 0.466 and 0.216, respectively), indicating the precision and reliability of the models (i.e., the model fits the data adequately). Thus, the regression models of total FA yields and the selected omega-3 FA yields established in this study were predictive, quantitative, and evaluative, with relatively satisfactory correlations between responses and process variables (i.e., the model not only captures the general trend but also adheres well to the finer structure within the data). Furthermore, the linear effects of pressure and mass ratio of co-solvent to feedstock, the square effects of pressure, and the interactions between temperature and mass ratio of co-solvent to feedstock were considered to significantly impact the total FA yields, while only the quadratic effects of pressure were regarded as a significant model term for the selected omega-3 FA yields (Table S3).

**Figure 2:**
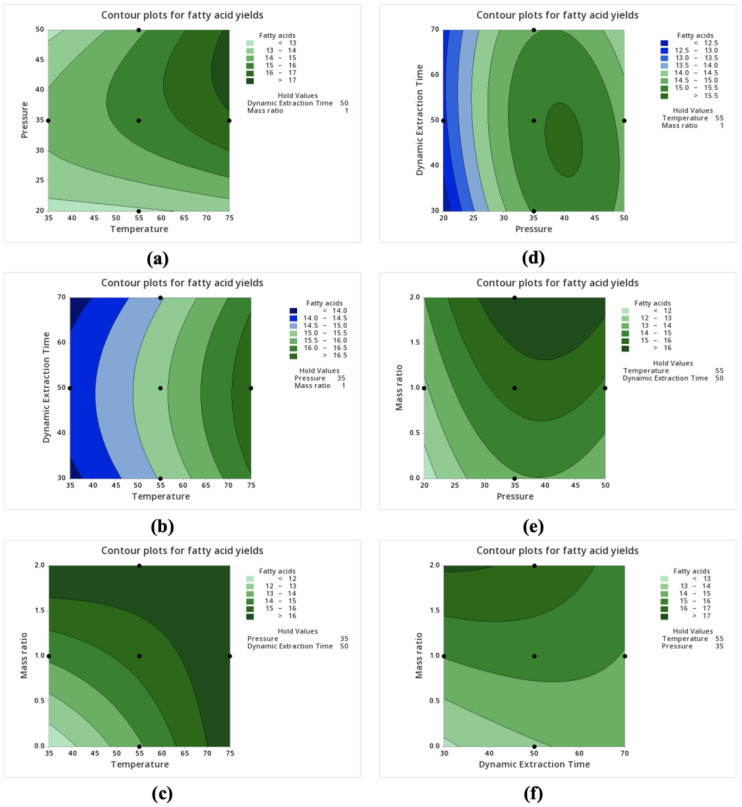
Contour plots for FA yields (g/100 g of feedstock on a dry weight basis) from scCO_2_ extraction of hot-air-dried *C. frondosa* viscera. Non-targeted variables were maintained at their median values, with the temperature at 55 °C, pressure at 35 MPa, dynamic extraction time at 50 min, and the co-solvent/feedstock mass ratio at 1.

**Figure 3:**
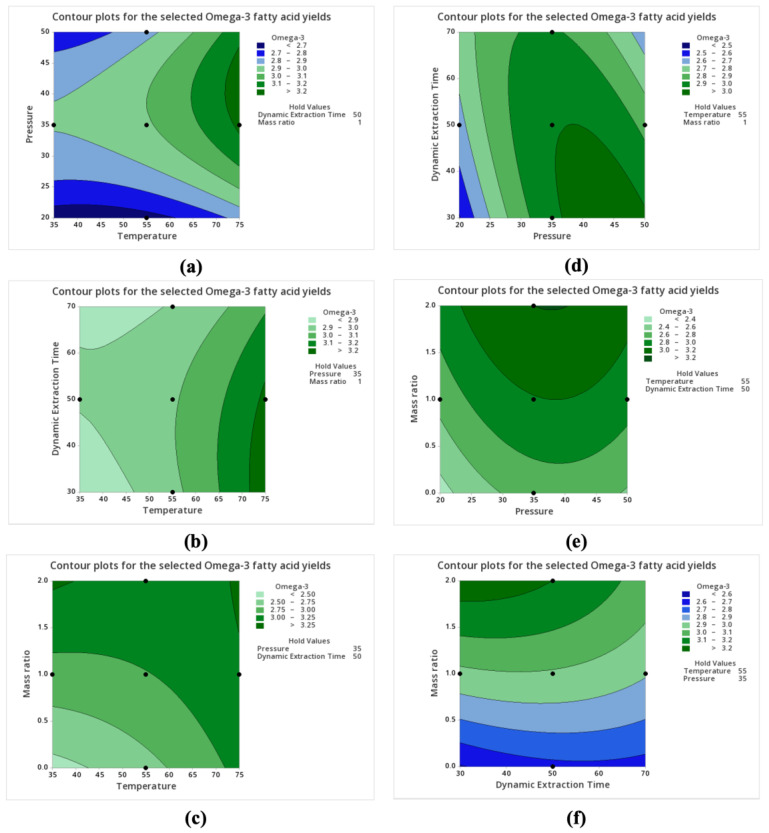
Contour plots for the selected omega-3 FA yields (g/100 g of feedstock on a dry weight basis) from scCO_2_ extraction of hot-air-dried *C. frondosa* viscera. Non-targeted variables were maintained at their median values, with the temperature at 55 °C, pressure at 35 MPa, dynamic extraction time at 50 min, and the co-solvent/feedstock mass ratio at 1.

Maximum yields of over 17 g of FAs and more than 3.2 g of EPA and DHA in 100 g of samples on a dry weight basis could be obtained at a temperature of about 73–75 °C and a pressure of approximately 36–47 MPa with constant dynamic extraction time and co-solvent/feedstock mass ratio (Figure 2a and Figure 3a). Pressure and temperature directly affect the density of the supercritical fluid, so higher pressures and higher temperatures might improve the solvating power of the supercritical fluid for FAs, leading to an increase in extraction yields. Moreover, higher temperatures enhance the intensive thermal motion of compounds, allowing easier transfer from samples to the supercritical solvents and thus increasing the solubility of FAs and contributing to higher extraction yields.

Increasing temperature leads to an increase in FA yields at a wide range of dynamic extraction times (Figure 2b). The highest EPA and DHA yields were obtained with increasing temperature, so long as the dynamic extraction time was shorter than about 52 min (Figure 3b). However, as the dynamic extraction time increased to 70 min at 75 °C, the selected omega-3 FA yields plateaued. The results re-emphasize that the higher temperature selection facilitates better solubility, improved kinetic properties of the solvent, and efficient phase separation, all contributing to the enhanced performance of the extraction process.

Increasing the co-solvent/feedstock mass ratio resulted in increases in extraction yields (Figure 2c,e,f, as well as Figure 3c,e,f). The addition of co-solvent ethanol increased the solubility of polar FAs and lipidic compounds (e.g., phospholipids) in scCO_2_ by enhancing the polarity and penetration of scCO_2_. Therefore, the extraction yields reached the maximum when the co-solvent/feedstock mass ratio was 2.

Both total FA yields and selected FA yields reached clear maxima in response to pressure and dynamic extraction time. For total FA yields, the maximum occurred at pressures from ~40 to 48 MPa and dynamic extraction times from ~38 to 52 min (Figure 2d). The highest EPA and DHA yields could be found when pressure was higher than about 36 MPa while the dynamic extraction time was simultaneously shorter than approximately 50 min (Figure 3d). The shorter dynamic extraction time giving maximum FA yields might be attributed to the quick saturation of the solvent capacity in the static extraction process dissolving most FAs and the equilibrium state of the extraction system in which continuing the extraction process does not significantly increase the yield but rather just maintains the equilibrium concentration of the extract in the solvent.

#### 2.1.2. Optimization of FA Yields

Comparing the three scenarios (maximum FAs, maximum selected omega-3 FAs, and maximum all responses, Table S4), the temperature of 75 °C, pressure of 45 MPa, static extraction time of 20 min, dynamic extraction time of 30 min, and mass ratio of ethanol/feedstock of 2:1 were found as the ideal setting for maximizing yields of all responses. Nevertheless, in pursuit of economic benefits, streamlining production plays an important role at the industrial level. Extraction without introducing co-solvents is the preference to avoid an additional extract purification process. Thus, the mass ratio of co-solvent/feedstock held at a value of 0 should also be considered.

Experiments under the optimal conditions with/without the co-solvent that maximize yields of FAs and the selected omega-3 FAs were carried out in quadruplicate (Table 1). The experimental results revealed the differences between FA yields and the selected omega-3 yields from these two suggested conditions were statistically significant. The statistical results were consistent with the ANOVA of the generated models, in which the linear term related to the co-solvent-to-feedstock mass ratio was statistically significant in the FA regression model. Total FA yields and selected FA yields were 9.72% and 17.17% higher, respectively, when co-solvent was added than without. The acquired experimental yields of total FAs and the selected omega-3 FAs all fell in the 95% predicted intervals; thus, good agreement between experimental values and model-determined values was obtained, reinforcing that the generated models are reliable. Moreover, the weight percent of the selected omega-3 FAs in the total FAs under the optimum with the co-solvent was higher at 20.71 ± 0.45% compared to without the co-solvent (19.40 ± 0.12%) with *p* < 0.05. Therefore, from the perspective of yields and composition, the optimal condition was a temperature of 75 °C, pressure of 45 MPa, dynamic extraction time of 30 min, and mass ratio of ethanol/feedstock of 2:1, but to make a decision for the incorporation of co-solvent in actual practice, a life cycle assessment and a techno-economic analysis of the whole process would be required.

The highest FA yields and selected omega-3 FA yields were obtained under the temperature of 75 °C. Although a study based on conventional cooking and pressing method mentioned EPA and DHA were significantly degraded at 50 °C or above [30], the experimental design allowed tuning to the optimized temperature at 75 °C, implying scCO_2_ might protect the extract from severe degradation that normally occurs in the conventional extraction. Létisse et al. (2006) also found a higher temperature of 75 °C yielded more EPA and DHA without degradation [31].

### 2.2. Comparison between Conventional and ScCO_2_ Methods

Conventional extraction based on the Bligh and Dyer method and the in situ transesterification was conducted, and the extraction yields of total FAs and the selected omega-3 FAs were compared with those of scCO_2_ extraction under the optimal conditions (Table 2). Total FA yields with scCO_2_ extraction of *C. frondosa* viscera were comparable with those from in situ transesterification and the ultrasonic-assisted in situ transesterification, while selected omega-3 FA yields with scCO_2_ extraction were comparable to those of the ultrasonic-assisted Bligh and Dyer method and in situ transesterification. Overall, the processing of hot-air-dried *C. frondosa* viscera using in situ transesterification and scCO_2_ extraction led to similar yields of total FAs, EPA, and DHA. However, the Bligh and Dyer method extracted significantly more crude lipids (39.66 ± 2.60%, over 10% more of crude lipids obtained from scCO_2_ extraction), which might be due to the co-extraction of some untargeted compounds (sterols, glycolipids, sugars and fat-soluble compounds, like carotenoids and some vitamins) and the limitations in recovering fatty-acid-rich lipid fractions, highlighting the higher selectivity of scCO_2_ extraction for fatty-acid-rich sea cucumber lipids. Therefore, scCO_2_ extraction is an ideal greener alternative to the conventional Bligh and Dyer method. Notably, the ultrasonic pre-treatment showed improvements in the conventional extraction of FAs. That might be because ultrasonication disrupted cells, resulting in a more complete extraction of lipids. Thus, developing more efficient pre-treatment (i.e., ultrasonic probe, microwave) in scCO_2_ extraction may further enhance the extraction of lipids and eliminate the use of co-solvents. On the other hand, the ultrasonic-assisted in situ transesterification gave greater FA yields than the Bligh and Dyer method followed by transesterification. The result was also supported by Cavonius et al. (2014) and Dickey et al. (2002), who compared direct transesterification with the Bligh and Dyer method through the determination of FA content in several microalgae species and striped bass tissues, respectively, and found the direct method gave greater FA yields [32,33].

EPA, palmitoleic acid (16:1n-7), and 12-methyltetradecanoic acid (12-MTA) were the major FAs. The highest proportions of EPA were achieved with the ultrasonic-assisted Bligh and Dyer method (22.49% in total FAs), while the greatest proportions of 12-MTA were found with scCO_2_ extraction and in situ transesterification (24.17% and 22.06% in total FAs) (Table S5). Zhong et al. (2007), Mamelona et al. (2010), Abuzaytoun et al. (2022), and Liu et al. (2021) reported similarly high proportions of EPA in *C. frondosa* (~20% in total FAs) [6,7,16,17]. Only a few studies have reported 12-MTA in *C. frondosa* and its by-products, varying from 11.79% to 21.27% in the total FAs [17]. 12-MTA can be used for tumor cell treatment individually or in combination with other nutritional or anticancer compounds through inducing cell-apoptosis-related metabolites or activities in cancer proliferation [34]. Our method yielded a significantly higher proportion of 12-MTA (~24.17% in the total FAs) compared to the Bligh and Dyer method (~21.35% in the total FAs), suggesting that optimal scCO_2_ extraction can serve as a reliable method for preparing 12-MTA with further potential health applications.

Studies point out that an excessive intake of omega-6 FAs has been widely observed in Western diets, leading to a higher ratio of omega-6/omega-3 FAs (15-20:1) [35]. Excess dietary omega-6 FAs are one of the main causes of inflammation in human bodies, while omega-3 FAs can inhibit inflammation by limiting the metabolism of omega-6 FAs and the generation of associated inflammatory substances [15]. A recommended ratio of omega-6/omega-3 FA was reported as 1-5:1 [36]. In *C. frondosa* viscera, the ratio of omega-6/omega-3 was approximately 0.05, which could balance the extra omega-6 to achieve a proper ratio of omega-6/omega-3 FAs. Therefore, *C. frondosa* viscera also possess great marketable features for commercializing as a natural source of nutritional supplements.

### 2.3. Effects of Pre-Treatments on FA Yields

#### 2.3.1. Effects of Different Drying Methods

Fresh, freeze-dried, and hot-air-dried samples were subjected to scCO_2_ extraction under the optimal conditions. Pre-treatments affected responses, with freeze-dried samples yielding significantly more total FAs and selected omega-3 FAs (Table 3). Fresh samples without pre-treatment gave the lowest yields. The results were consistent with the FA contents of those samples obtained from the ultrasonic-assisted in situ transesterification (Table S6). The moisture contents of fresh, freeze-dried, and hot-air-dried samples were different at ~83, 3, and 6 wt.%, respectively (Table S6), likely explaining the variation in FA yields among different pre-treatment types.

When compared to yields from the maximum FA recovery obtained from the ultrasonic-assisted in situ transesterification of freeze-dried samples, almost all FAs (96.40%) and similar amounts of EPA and DHA were recovered in scCO_2_ extraction of freeze-dried samples (Table 3); however, scCO_2_ extraction of hot-air-dried samples showed reduced yields with 73.55% of total FAs and 81.67% of selected omega-3 FAs recovered. Yields were far lower in the fresh samples with only 23.21% of total FAs and 32.23% of the selected omega-3 FAs recovered. These results are almost surely attributed to higher moisture content in the fresh samples hindering the penetration of scCO_2_ for FA extraction. The very similar content of selected omega-3 FAs obtained from ultrasonic-assisted in situ transesterification of the freeze-dried samples (4.14 ± 0.06 g/100 g samples on a dry weight basis) and that from scCO_2_ extraction of the freeze-dried samples (4.27 ± 0.29 g/100 g samples on a dry weight basis) allows us to conclude that scCO_2_ extraction of the freeze-dried samples recovered the same amount of EPA and DHA that can be achieved with chemical extraction.

The comparison of yields and recovery efficiencies suggests that freeze-dried sea cucumber samples are preferable to fresh and hot-air-dried samples when maximum FAs recovery is the goal. It is likely that the presence of water creates surface charges on the cells that interfere in the establishment of contact between cells and CO_2_ or other extraction solvents [37]. Water molecules are polar, possessing partial positive and negative charges, which enables them to interact with other polar substances and ions to form hydration shells. This polar nature of water molecules results in the formation of a structured layer around cellular components, which can interfere with the contact between cells and solvents like CO_2_. In some studies related to scCO_2_ extraction of lipids from algae, researchers found an increase in the biomass water content up to 20 wt.% increased the global extraction (extraction of pigments, water, and oil) and did not restrict the extraction process but had no influence on oil extraction yields [29,38]. However, our study found that a moisture content of over 80 wt.% had a detrimental effect on the extraction process, leading to lower FA extraction yields with lower water content, giving higher extraction efficiency. Thus, it implies that the moisture content of samples should be controlled under 20 wt.%, and it will likely be necessary to remove extra water before extracting fresh material with high moisture contents (e.g., squeezing the samples to get rid of the water). Furthermore, freeze-drying along with grinding may be more effective in cell disruption, giving higher extraction yields, while hot-air drying may preserve cells better since relatively lower extraction yields were given. As a result, freeze-drying followed by grinding enhances scCO_2_ penetration within the biomass and sequentially improves FA extraction from *C. frondosa* viscera, although it is not a commercially viable process.

The FA proportions of hot-air-dried and freeze-dried viscera after scCO_2_ extraction were similar to each other but significantly different than those of the fresh samples (Table S7). Again EPA, 16:1n-7, and 12-MTA were the primary FAs, with EPA as the predominant FA in fresh viscera, accounting for 24.59% of total FAs, and 12-MTA as the predominant FA in hot-air-dried and freeze-dried samples, comprising 24.17% and 26.76% of total FAs, respectively. Hot-air-dried and freeze-dried samples also contained notable proportions of 19.83% and 18.62% of EPA, respectively. DHA was also present in all samples but at a very low amount of 1.01–1.46%. Compared with dried samples, fresh samples contained significantly higher portions of saturated FAs, monounsaturated FAs, and PUFAs (including omega-3) in the total but lower proportions of branched-chain FAs, including 12-MTA.

**Figure 4:**
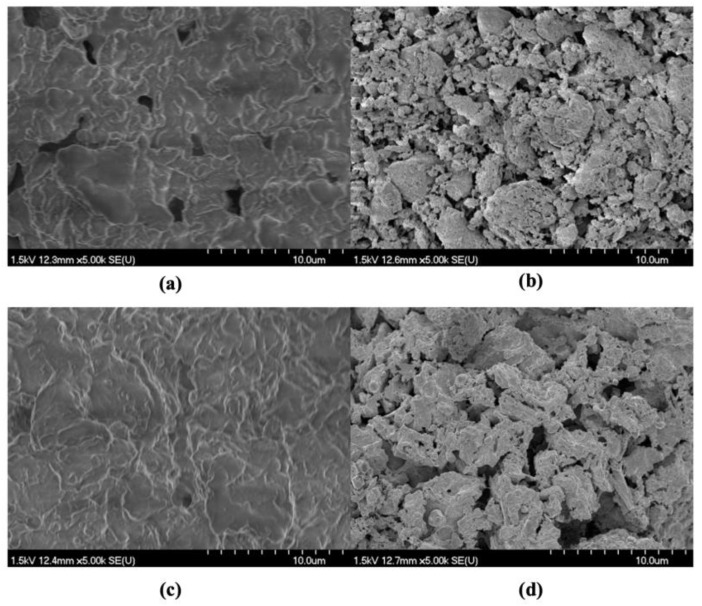
SEM images of hot-air-dried *C. frondosa* viscera (**a**) before and (**b**) after scCO_2_ extraction under the optimal conditions and freeze-dried viscera (**c**) before and (**d**) after extraction under the optimal conditions.

To obtain a better understanding of the samples undergoing different drying methods, SEM was employed to analyze hot-air-dried and freeze-dried samples before and after scCO_2_ extraction under the optimal conditions (Figure 4). The surface structure of the freeze-dried material (Figure 4c) showed more obvious cracks and alternations than the air-dried material, indicating that freeze-drying led to the alteration of the biomass surface. Taher et al. (2014) found the surface structure of samples was cracked and altered by freeze-drying pre-treatment in comparison with sun-dried samples [39]. Sample rigidity was greater for hot-air-dried samples because of the low drying temperature and higher moisture content [40]. The lower rigidity of freeze-dried samples likely made it easier for them to be broken into smaller particles under the stream of scCO_2_, leading to a larger surface area and then further increasing the contact with extraction solvents. Therefore, freeze-drying may have improved the mass transfer within samples while also allowing scCO_2_ to penetrate the freeze-dried sample matrix more easily, enhancing lipid dissolution, and hence, the FA yields.

#### 2.3.2. Effects of Ethanol-Soaking

Kermanshahi pour et al. (2020) pointed out that processing ethanol-treated wet biomass (Thraustochytrids) led to a recovery of PUFAs comparable to those achieved in the processing of freeze-dried biomass [41]. To examine the feasibility of ethanol-soaking pre-treatment, fresh and hot-air-dried *C. frondosa* viscera were soaked into 95% ethanol, followed by scCO_2_ extraction under the optimum conditions (Table 3), showing that ethanol-soaking pre-treatment significantly increased the extraction yields of fresh viscera, achieving almost triple the yields obtained from fresh samples without ethanol-soaking. Immersing wet biomass in 95% ethanol exchanges its moisture through osmotic dehydration, enhancing extraction efficiency [42]. The hygroscopic property of ethanol allows it to absorb water, aiding in breaking down cell structures and increasing compound solubility, thus boosting yields. ScCO_2_ extraction of ethanol-soaked fresh samples, hot-air-dried samples, and ethanol-soaked hot-air-dried samples led to a similar recovery of the selected omega-3 FAs (a higher portion of omega-3 FAs in ethanol-soaked fresh sample), which suggested the widely used hot air dryer in the industry could be replaced by an ethanol-soaking pre-treatment to achieve the energy-saving purpose when processing *C. frondosa* viscera for EPA and DHA extraction. Notably, ethanol-soaking did not significantly improve the extraction of total FAs from hot-air-dried viscera. That might be because the ethanol-soaking was not able to exchange a very small amount of moisture and did not further modify the microstructure of hot-air-dried samples. Although ethanol-soaking pre-treatment enhanced the extraction of total FAs from fresh samples, freeze-drying still performed better than ethanol-soaking of samples. That might be attributed to the better cell disruption effect on the freeze-dried samples, in which the lowest moisture content influences the robustness of the cells and augments the efficiency of cell disruption. Importantly, ethanol-soaking pre-treatment did not change the PUFA proportions in the fresh materials, but it gave a higher proportion of 12-MTA compared with fresh materials and enhanced the PUFA proportions in the extracts in contrast to freeze-drying and hot-air drying (Table S7).

Despite freeze-drying leading to a higher product yield compared to hot-air drying and ethanol-soaking of fresh material, it is energy-intensive, consuming approximately four to ten times more energy than hot-air drying [43]. This substantial increase in energy consumption contributes to higher environmental impacts, a crucial consideration in today’s ecologically conscious market. Additionally, scaling up freeze-drying for industrial applications presents considerable difficulties, impeding its practicality and widespread adoption in various industries; moreover, it is inherently costly, posing financial challenges to industries. Hot-air drying is also energy intensive but typically consumes less energy than freeze-drying. These limitations highlight the need for innovative pre-treatments, such as ethanol-soaking of fresh material, which is significantly less energy-intensive compared to freeze-drying.

## 3. Materials and Methods

### 3.1. Materials and Chemicals

Frozen fresh and hot-air-dried *C. frondosa* viscera were provided by AKSO Marine Biotech Inc. *C. frondosa* was harvested in the Fishing Area FAO 21 and kept frozen until the viscera were separated from other body parts. The harvested *C. frondosa* was thawed and washed with seawater, and then eviscerated. The collected viscera were dried under hot air at 40–45 °C for 48 h. After processing, the frozen fresh viscera and the hot-air-dried ones were stored in a cooler and delivered to the Biorefining and Remediation Laboratory at Dalhousie University.

Ultra-high-purity liquid carbon dioxide, nitrogen, helium, air, and hydrogen were purchased from Praxair (Dartmouth, NS, Canada). Liquid carbon dioxide was used as the solvent for scCO_2_ extraction. Internal standard methyl nonadecanoate (C19:0 methyl ester) and external standards 37 FAME mix standard and PUFA No.3, butylated hydroxytoluene (BHT), sodium chloride (NaCl), potassium chloride (KCl), sodium sulphate (Na_2_SO_4_), anhydrous methanol, agent grade acetyl chloride, Whatman #1 filter paper, and glass wool were supplied by Sigma Aldrich Ltd. (Oakville, ON, Canada). ACS-grade toluene, sodium bicarbonate (NaHCO_3_), GC-grade hexane, and GC-grade chloroform were purchased from VWR International Ltd. (Mississauga, ON, Canada). Ninety-five percent ethanol was purchased from Tupper Medical Store at Dalhousie University (Halifax, NS, Canada). All chemicals were used as received.

### 3.2. Methods

#### 3.2.1. Sample Preparation

To evaluate the effects of different pre-treatments on scCO_2_ extraction, frozen fresh sea cucumber viscera were freeze-dried using a bench-top freeze dryer (Labconco Freezone 2.5 L Bench-top Freeze Dry System, Kansas City, MO, USA). The freeze-drying method was adapted from Liu et al. (2021) [16]. Fresh *C. frondosa* viscera were separated into several portions, placed in 20 mL tubes for freezing overnight at −20 °C, and then transferred to freeze dryer flasks. The flasks were loaded onto the freeze dryer, and the temperature and pressure were set at ~−87 °C and 0.014 mBar for two days.

Fresh and dehydrated samples were ground in an electric grinder (Shardor Technology Ltd., Changsha, Hunan, China) and sieved using an 18-mesh-sized sieve (<1 mm). The ground samples were hermetically sealed in airtight zippered freezer bags and stored at −20 °C until required.

#### 3.2.2. Moisture Content Determination

Moisture content was determined gravimetrically using a vacuum oven (Across International, Livingston, NJ, USA) according to a method modified from AOAC (2006) [44]. Approximately 1.5 g samples in aluminum dishes were weighed on an analytical balance (Mettler Toledo AT261 Analytical Balance, Columbus, OH, USA) and dried at 105 °C until constant mass was achieved. The measurement was carried out in triplicate for each sample.

#### 3.2.3. The Bligh and Dyer Method

Crude lipids *of C. frondosa* viscera were extracted according to a modified Bligh and Dyer method [45,46]. Approximately 1.5 g hot-air-dried samples in quadruplicate were weighed, followed by the addition of 6 mL of distilled water to make up to 80% of the moisture content. Then, 7 mL of chloroform with 0.01% BHT and 15 mL of methanol (chloroform/methanol/water (*v*/*v*/*v*) = 1:2:0.8) were also added. The mixture was vortexed vigorously until the mixture formed a single phase. The well-homogenized mixture was filtered, and then the tissue residue, the tube, and the filter paper were rinsed with 7.5 mL of chloroform. The filtrate was mixed with 7.5 mL of 0.88% KCl solution to induce layer separation. The resulting proportions of chloroform/methanol/water (*v*/*v*/*v*) were 2:2:1.8 in the final mixture. The chloroform–methanol layer was collected using centrifugation. Anhydrous Na_2_SO_4_ was placed onto the filter paper cone in a funnel to absorb extra moisture in the extracts. Following filtration, the filter paper with Na_2_SO_4_ was rinsed using 3–4 mL of chloroform. The lipid-rich chloroform was recovered and evaporated using a rotary evaporator (Buchi, Switzerland) at 45 °C, 258 bar. The extracts were further dried under nitrogen, weighed, and stored at −20 °C. The extracts were considered as the global extracts, determined as the ratio between the mass of the extract and the mass of biomass.

An improved ultrasonic-assisted Bligh and Dyer method was employed. After the addition of water, chloroform, and methanol to the ratio of 1:2:0.8 (*v*/*v*/*v*), the mixture was covered with a stream of nitrogen and placed in an ultrasonic water bath (Branson 2510 sonicator, Brookfield, CT, USA) for 10 min. The subsequent steps were consistent with the previous description.

#### 3.2.4. ScCO_2_ Extraction

The scCO_2_ extraction apparatus (SFE-110, Supercritical Fluid Technologies Inc., Newark, DE, USA) comprised a CO_2_ cylinder, a CO_2_ pump, a co-solvent pump, a heating chamber, a sample vessel, collection vials, and a flow meter. Approximately 1.5 g of dried *C. frondosa* viscera, or approximately 8.9–9.0 g of fresh samples with a dry weight of 1.5 g along with 95% ethanol as co-solvent, was loaded directly in a 10 mL sample vessel. In the case of fresh viscera, the co-solvent ethanol was introduced into the vessel through the co-solvent pump. The tightly sealed vessel was connected to the system. The extraction procedure included static and dynamic phases. The static stage was recorded once the temperature and pressure achieved the set point in the closed system. This step allows contact between the sample and the supercritical solvent. After the pre-determined time of static soaking, the static/dynamic valve was opened, and the restrictor valve was slightly adjusted to ensure scCO_2_ was continuously drained through the vessel at approximately 10 mL/min. During the dynamic extraction, the extraction time was recorded as well. Two pre-weighed vials (a collection vial and a trap vial) were used to collect the extracts. After extraction, the system was vented until the pressure of the vessel dropped to atmospheric pressure and the temperature reached room temperature. The vessel was opened to remove the residues and then reconnected to the system for washing. Ethanol (95%) was purged using the co-solvent pump to rinse the line and the vessel to collect the remaining extract. The washing solution was evaporated in a rotary evaporator until no solvent was trapped. The washout extract after evaporation was added to the original extract, which was regarded as the global extract. The global extract was weighed, covered with an inert atmosphere of nitrogen, and stored at −20 °C.

To verify whether ethanol-soaking pre-treatment can improve the extraction yields of hot-air-dried and fresh samples, approximately 1.5 g of hot-air-dried *C. frondosa* viscera and specific quantities of fresh samples with masses equivalent to 1.5 g of dry weight (~8.9–9.0 g) were soaked in 3 g and 6 g of 95% ethanol for two hours, respectively. The rest of the ethanol co-solvent for fresh sample trials (~12 g) was pumped into the system through the co-solvent pump and then extracted under the optimum conditions following the method mentioned above.

#### 3.2.5. Transesterification after Extraction

FAs were converted into FAMEs using methanolic HCl transesterification, which was adapted from Crompton and Dunstan (2018) [47]. Toluene with 0.01% BHT was added to the lipid extract to achieve a 100 mg/mL concentration. Then, 1.5 mL of extract-rich toluene was transferred into a clean tube with dried methyl C19:0 FAME (10 mg/mL in hexane, 5 μL). FAMEs were formed by treating the mixture with 3 mL of 5% methanolic HCl at 100 °C for one hour. A 5% NaCl solution was added, and the top organic phases were exhaustively recovered with hexane. Excess acid was neutralized with 2% NaHCO_3_, and residual moisture was absorbed by anhydrous Na_2_SO_4_. FAMEs were then dried under a stream of nitrogen and stored at −20 °C.

#### 3.2.6. In Situ Transesterification

The in situ transesterification was adapted from Crompton and Dunstan (2018) [47]. In in situ transesterification, the methanolic HCl method was applied to four replicates of identical *C. frondosa* viscera (500 mg of dried samples, or approximately 2.98 g fresh samples with a dry weight of 500 mg) with dried C19:0 FAME (10 mg/mL in hexane, 5 μL) added as the internal standard. Before the addition of the methanolic HCl, the sample and 1.5 mL toluene containing 0.01% BHT were treated in an ultrasonic water bath for 10 min. The transesterification reaction was the same as described previously.

#### 3.2.7. FA Profiles in GC

The methyl esters obtained from transesterification were diluted using hexane and transferred to a pre-labeled amber GC vial covered with nitrogen. The external standards—37 FAME mix and PUFA No.3—were used as references for qualitative analysis. Gas chromatography equipped with a flame ionization detector (GC-FID, Agilent 7890B GC, Santa Clara, CA, USA) and DB-23 column was applied to analyze samples and standards. The carrier gas was helium, using a split ratio of 1:50. The detector and injector temperatures were 280 and 250 °C, respectively. The column was maintained at 50 °C for 1 min, raised from 50 to 215 °C at 25 °C/min, increased to 230 °C at 4 °C/min, and finally maintained at 230 °C for 5 min. One microliter of the methyl esters was injected into the column, and the FAs were identified by comparing the retention times with those of the methyl ester standards. The calculation of FA quantities in the feedstock, yields, and recovery efficiencies is presented in the Supplementary Materials (Text S1).

#### 3.2.8. Morphological Imaging

A Hitachi S-4700 FE scanning electron microscope (Chiyoda, Tokyo, Japan) was used to investigate morphological structure changes due to different pre-treatments. To avoid charge-up effects on the surface and promote the emission of secondary electrons, dried samples were mounted on aluminum stubs and coated with a gold–palladium alloy for 260 s, and 20-nanometer coated layers were obtained. The coated samples were scanned using the scanning electron microscope at an accelerating voltage of 1.5 kV.

#### 3.2.9. Experimental Design and Statistical Analysis

To optimize yields of omega-3 FAs from the scCO_2_ extraction of *C. frondosa* viscera, a preliminary screening was carried out using single-factor experiments by fixing all continuous variables except one. In the preliminary screening, the temperatures of 35, 55, and 80 °C, the pressures of 20, 35, and 50 MPa, the dynamic extraction times at 30, 60, 135, and 210 min, the static times at 0, 10, 20, and 30 min, and the co-solvent-ethanol-to-feedstock mass ratios at 0 (no co-solvent added), 1, and 2 were tested on hot-air-dried samples. The preliminary screening experiments were performed in duplicate. According to the results from the preliminary screening, the range of selected process variables was specified.

A response surface method, CCI design, with four parameters—temperature (35–75 °C), pressure (20–50 MPa), dynamic extraction time (30–70 min), and mass ratio of co-solvent to feedstock (0–2)—was created in Minitab 19 (Minitab Inc., State College, PA, USA). Each factor was examined at five levels: −α, −1, 0, +1, and +α (Table 4). The design included center points that were run 7 times, as well as 16 cube points and 8 axial points, resulting in 31 trials (Table S1). The total FA yields and the selected omega-3 FA (DHA and EPA) yields were evaluated as the response variables for optimization. The pre-treatments (fresh, hot-air-dried, freeze-dried ethanol-soaking) were considered as categorical variables and compared under the optimal conditions.

Overall, the RSM combines regression modeling and analysis of variance (ANOVA), resulting in a model (Equation (3)):

(3) $$Y=\beta_{0}+\sum_{i=1}^{k}\beta_{i}X_{i}+\sum_{i=1}^{k}\beta_{ii}{X_{i}}^{2}+\sum_{i=1}\sum_{i<j}\beta_{ij}X_{i}X_{j}$$

where *Y* represents the response, *X_i_* and *X_j_* are the independent variables, $\beta_{0}$ is the intercept coefficient, and $\beta_{i}$, $\beta_{ii}$, and $\beta_{ij}$ are the linear, quadratic, and interaction coefficients.

The independence assumption was met due to the randomization of the run orders. The significance of each model term was examined at an alpha level of 0.05. The determination coefficient (R^2^) and *p*-values of lack of fit were used to evaluate the goodness of the fits to the regression models. Additional runs at the optimum conditions were conducted to validate the model, and the experimental results were compared with the model prediction. Three-dimensional surface plots and contour plots were generated by varying two variables within the studied range and holding the other two consistent.

## 4. Conclusions

This is the first paper on scCO_2_ extraction of omega-3-FA-rich oils from *C. frondosa* viscera, combined with RSM, to explore the optimization of FA and omega-3 FA yields. The process under the temperature of 75 °C, pressure of 45 MPa, static extraction time of 20 min, dynamic extraction time of 30 min, and co-solvent-ethanol-to-feedstock mass ratio of 2:1 yielded 16.207 g of FAs and 3.378 g of the selected omega-3 FAs in 100 g of hot-air-dried samples, with approximately 74% of FAs and 82% of EPA and DHA recovered. The experimental yields all fell in the 95% predicted interval, revealing the generated regression models were applicable to yield prediction. ScCO_2_ extraction in this study resulted in yields comparable with or even higher than those produced from the Bligh and Dyer method, indicating that scCO_2_ extraction is an ideal alternative to conventional extraction with higher selectivity for FAs. With respect to the pre-treatments prior to scCO_2_ extraction, freeze-drying significantly improved the extraction yields, resulting in about 96.4% of FAs and almost all EPA and DHA being recovered. Moreover, ethanol-soaking pre-treatment of fresh samples prior to scCO_2_ extraction significantly improved the extraction yields of FAs, recovered a similar amount of EPA and DHA compared with hot-air drying, and increased the proportions of omega-3 FAs in the total FAs compared with dried samples. This innovative approach eliminates the use of toxic solvents, addressing concerns about residual organic solvents in nutraceuticals and pharmaceuticals. The findings underscore the potential of scCO_2_ extraction for enhancing the efficiency and sustainability of omega-3-FA-rich oil extraction from sea cucumber viscera.

## Figures and Tables

**Table 1:** Experimental yields of FAs and the selected omega-3 FAs (g/100 g of samples on a dry weight basis) from the hot-air-dried samples under optimal conditions with/without co-solvents added (*n* = 4, Mean ± SD). Values in the same column with different letters are significantly different at *p* < 0.05.

Optimal Conditions		FA Yields	Selected Omega-3 FA Yields
Temperature of 75 °C, pressure of 45 MPa, dynamic extraction time of 30 min, and co-solvent to feedstock mass ratio of 2:1	Experimental value	16.30 ± 0.66 ^a^	3.38 ± 0.20 ^a^
	Predicted value	18.06	3.47
	Predicted interval	(15.24, 20.87)	(2.67, 4.27)
	Recovery % ^1^	73.55	81.67
Temperature of 75 °C, pressure of 44 MPa, dynamic extraction time of 41 min, and co-solvent to feedstock mass ratio of 0:1	Experimental value	14.86 ± 0.08 ^b^	2.88 ± 0.03 ^b^
	Predicted value	16.92	3.14
	Predicted interval	(14.73, 19.11)	(2.52, 3.77)
	Recovery % ^1^	67.04	69.71

^1^: recovery efficiencies are calculated as the ratio of the experimental value to the maximum contents of FAs and the selected omega-3 FAs, which were obtained from the ultrasonic-assisted in situ transesterification of freeze-dried samples (22.17 g and 4.14 g/100 g of samples on a dry weight basis).

**Table 2:** A comparison of FA yields and selected omega-3 FA yields of hot-air-dried *C. frondosa* viscera (g/100 g of sample on a dry weight basis) that are obtained from the Bligh and Dyer method (*n* = 4, Mean ± SD), in situ transesterification (n = 4, Mean ± SD), and scCO_2_ extraction under the optimal condition (*n* = 4, Mean ± SD). Values in the same column with different letters are significantly different at *p* < 0.05.

Methods	FAs	Selected Omega-3 FAs
Bligh and Dyer	9.02 ± 1.55 ^a^	2.22 ± 0.34 ^a^
Ultrasonic-assisted Bligh and Dyer	13.59 ± 0.80 ^b^	3.16 ± 0.18 ^b^
In situ transesterification	15.94 ± 0.35 ^c^	3.72 ± 0.15 ^cd^
Ultrasonic-assisted in situ transesterification	17.43 ± 0.20 ^c^	3.92 ± 0.05 ^d^
scCO_2_ extraction	16.30 ± 0.66 ^c^	3.38 ± 0.20 ^bc^

**Table 3:** Experimental yields (g/100 g of samples on a dry weight basis) of the total FAs and the selected omega-3 FAs under the proposed optimal condition of scCO_2_ extraction (*n* = 4 for all except *n* = 3 for EtOH soaking, Mean ± SD). Samples underwent different pre-treatments, and the Tukey test compared the experimental yields at a 95% confidence level. Values in the same column with different letters are significantly different at *p* < 0.05.

Pre-Treatment	FA Yields	Recovery % ^1^	Selected Omega-3 Yields	Recovery % ^1^
Fresh samples	5.14 ± 0.48 ^a^	23.21	1.33 ± 0.18 ^a^	32.23
Hot air dry	16.30 ± 0.66 ^b^	73.55	3.38 ± 0.20 ^b^	81.67
Freeze dry	21.37 ± 1.06 ^c^	96.40	4.27 ± 0.29 ^c^	~100
Fresh + EtOH	13.68 ± 0.64 ^d^	61.73	3.47 ± 0.09 ^b^	83.80
Hot air dry + EtOH	16.56 ± 0.32 ^b^	74.72	3.69 ± 0.11 ^b^	89.19

^1^: recovery efficiencies are calculated as the ratio of the experimental value to the maximum contents of FAs and the selected omega-3 FAs, which were obtained from the ultrasonic-assisted in situ transesterification of freeze-dried samples (22.17 g and 4.13 g/100 g of samples on a dry weight basis).

**Table 4:** Process variables and their coded levels.

Process Variables	Levels				
	−α	−1	0	+1	+α
Temperature (°C)	35	45	55	65	75
Pressure (MPa)	20	27.5	35	42.5	50
Dynamic extraction time (min)	30	40	50	60	70
Ratio of co-solvent to feedstock (*w*/*w*)	0	0.5	1	1.5	2

## Data Availability

The original contributions presented in the study are included in the article/Supplementary Materials; further inquiries can be directed to the corresponding author.

## Supplementary Materials

The following supporting information can be downloaded at https://www.mdpi.com/article/10.3390/md22080366/s1: Text S1: FA profiles in GC; Text S2: preliminary screening (single-factor experiments) [21,48]; Figures S1–S5: preliminary screening results; Figure S6: actual vs. predicted values of yields of FAs and the selected omega-3 FAs; Figure S7: the optimum of scCO_2_ extraction with co-solvent for maximum FAs, maximum the selected omega-3 FAs, and maximum all responses; Figure S8: the optimum of scCO_2_ extraction without co-solvent for maximum FAs, maximum the selected omega-3 FAs, and maximum all responses; Table S1: central composite experimental design (inscribed) for scCO_2_ extraction of omega-3 fatty acids (FAs) from sea cucumber viscera; Table S2: the CCI design of process variables with corresponding experimental responses (yields of the total FAs and the selected omega-3 FAs), predicted values (predicted yields of the total FAs and the selected omega-3 FAs), and recovery efficiencies (compared with the maximum FA contents obtained from in situ transesterification of freeze-dried samples); Table S3: analysis of variance (ANOVA) for response surface models of FA yields and the selected omega-3 FA yields; Table S4: optimal conditions of scCO_2_ extraction of *C. frondosa* viscera (hot-air-dried) for maximum FAs and the selected omega-3 FAs; Table S5: the FA profiles of the global extract of hot-air-dried *C. frondosa* viscera obtained from the ultrasonic-assisted Bligh and Dyer method, the ultrasonic-assisted in situ transesterification, and scCO_2_ extraction; Table S6: ultrasonic-assisted in situ transesterification of fresh, hot-air-dried, and freeze-dried samples with contents of FAs and omega-3 FAs and moisture; Table S7: the FA profiles of the global extract of *C. frondosa* viscera obtained from scCO_2_ extraction under the optimal condition; viscera underwent different pre-treatments.
