# Supplementary material for: Extraction of Omega-3 Fatty Acids from Atlantic Sea Cucumber (Cucumaria frondosa) Viscera Using Supercritical Carbon Dioxide
Source: Mar Drugs. 2024 Aug 12;22(8):366. doi: 10.3390/md22080366 (PMC11355705; doi:10.3390/md22080366)
Supplement: Supplementary file 1 [file marinedrugs-22-00366-s001.zip › marinedrugs-3134003-supplementary.pdf]

Supplementary Materials

Extraction of Omega-3 Fatty Acids from Atlantic Sea Cucumber (*Cucumaria frondosa*) Viscera Using Supercritical Carbon Dioxide

Jianan Lin[a], Guangling Jiao[b], Marianne Su-Ling Brooks[c], Suzanne M.Budge[c], Azadeh Kermanshahi-pour[a*]

[a] Biorefining and Remediation Laboratory, Department of Process Engineering and Applied Science, Dalhousie University, Halifax, Nova Scotia B3J 1B6, Canada; jianan.lin@dal.ca; azadeh.kermanshahipour@dal.ca

[b] AKSO Marine Biotech Inc., Hacketts Cove, Nova Scotia B3Z 3K7, Canada

[c] Department of Process Engineering and Applied Science, Dalhousie University, Halifax, Nova Scotia, B3H 4R2, Canada

[*] Correspondence: azadeh.kermanshahipour@dal.ca; Tel.: +1-902-494-6076

Text S1: FA profiles in GC

FA amount in the sample was quantified by comparison of peaks from GC analysis between a known amount of added methyl C19:0 (**Equation S(1) & S(2)**):

$$Amount\ of\ a\ fatty\ acid = \frac{peak\ area_{a\ fatty\ acid} \times amount_{methyl\ C19:0}}{peak\ area_{\ methyl\ C19:0}} \tag{1}$$

$$Amount\ of\ total\ fatty\ acids = \frac{\Sigma(peak\ area_{each\ fatty\ acid} \times amount_{methyl\ C19:0})}{peak\ area_{\ methyl\ C19:0}} \tag{2}$$

The FA yields on the dry weight basis (g/100 g of feedstock used; dry weight basis means the moisture portion should be subtracted from the original sample weight) were calculated as **Equation S(3)**:

$$Fatty\ acid\ yields = \frac{Amount\ of\ total\ or\ certain\ fatty\ acids}{Amount\ of\ biomass\ used\ on\ the\ dried\ weight\ basis} \tag{3}$$

Results of FA composition were expressed as the percentage of an FA in the total FAs. Furthermore, comparing the total FAs with the FAs recovered by *in-situ* transesterification, the recovery efficiency (% recovery) was calculated as **Equation S(4)**:

$$Fatty\ acids\ recovery\ efficiency = \frac{Fatty\ acid\ yields\ of\ scCO_2\ extraction}{Fatty\ acid\ yields\ of\ conventional\ extraction} \times 100\% \tag{4}$$

Text S2: Preliminary screening (single-factor experiments)

Single-factor experiments were employed to identify important factors that should be considered in a response surface design for $scCO_2$ extraction of FAs from *Cucumaria frondosa* (*C. frondosa*) viscera. The yields of the total FAs and selected Omega-3 FAs versus potential factors were shown in **Figure S1 – Figure S5**.

Figure S1 showed as temperature increased yields of FAs changed marginally with no curvature. The one-way ANOVA suggested temperature had no significant effect on responses (p-values > 0.05).

In **Figure S2**, the change of yields as the function of pressure indicated FA yields increased and then decreased as pressure increased. The one-way ANOVA suggests that pressure had significant effects on all the responses (p-values < 0.05). Also, the Tukey pairwise comparisons supported a curvature for FA yields at the selected pressure range. An increase in pressure enhances the fluid density of scCO_2 and thereby strengthens the contact between scCO_2 and samples, whereas the decrease in extraction yields with increasing pressure after a specific point might be attributed to the increasing non-polar nature of the scCO_2 [1,2].

It was observed that by increasing the static extraction time from 0 to 10 to 20 to 30 min, the extracts eluted immediately when the static/dynamic valve and the restrictor valve were opened after the 20 min of soaking. **Figure S3** demonstrated yields did not change in response to increasing static extraction time from 20 to 30 min. Statistical analysis also supported this conclusion, revealing no significant changes in yields of FAs from 20 to 30 min of static time (p-values > 0.05). Thus, the static time of 20 min allows for sufficient contact between samples and scCO_2.

Figure S4 (a)-**(c)** exhibited dynamic time, which did not significantly impact the extraction yields (p-values > 0.05), although as dynamic time increased, responses rose. Dynamic extraction time might need to be adjusted based on the actual sample weights used; as such, to design more complex models with better predictors (allowed to cover insignificant effects), dynamic extraction time from 30 min to 75 min was selected as a variable to explore the most suitable option.

Figure S5 displayed trends of the responses as the mass ratio of co-solvent to feedstock increased. One-way ANOVA revealed with the mass ratio of co-solvent to feedstock increased from 0:1 to 2:1, the yield of selected omega-3 FAs increased significantly (p-value < 0.05), whereas a near-marginal significant effect on the total FA yields was found (p-value = 0.055 > 0.05). Nevertheless, the Tukey pairwise comparisons indicated that increasing the mass ratio of co-solvent to feedstock from 1:1 to 2:1 affected the total FAs yields (p-value < 0.05), and there was no significant difference in increasing the mass ratio from 2:1 to 4:1. That might be because of increasing the polarity of $scCO_2$ due to the presence of ethanol.

The solubility of target compounds depends on $scCO_2$ density, which is as a function of temperature and pressure, and the polarity of extraction solvents; as such, the temperature and the mass ratio of co-solvent to feedstock may have squared effects or interactions with other factors.

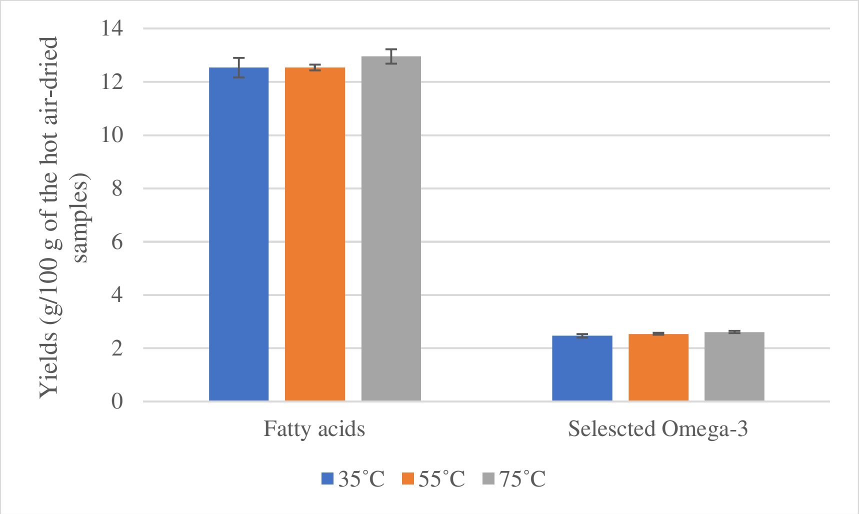

Figure S1. Effects of temperature (35, 55, and 75 ˚C) on yields of FAs and selected omega-3 FAs at 50 MPa of pressure, 20 min of static extraction time, 60 min of dynamic extraction time, and 0:1 of co-solvent to feedstock ratio. Error bars show the standard error of the mean for duplicate samples, n=2.

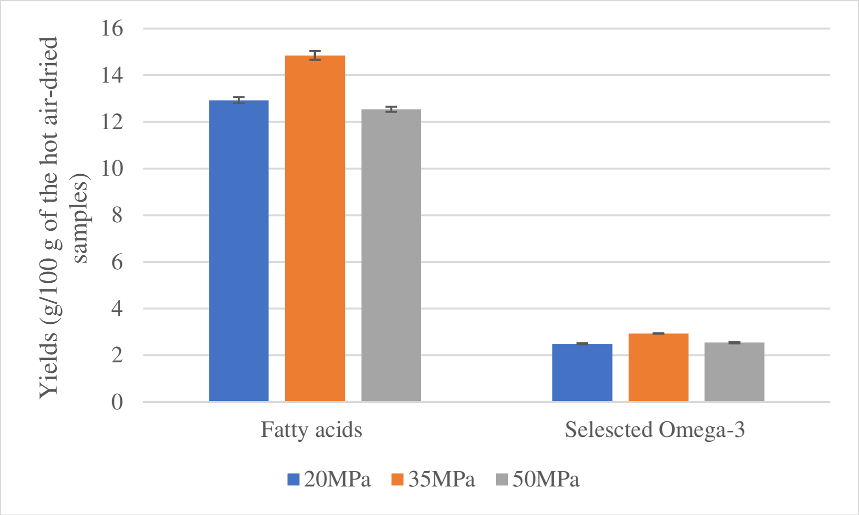

Figure S2. Effects of pressure (20, 35, and 50 MPa) on yields of FAs and selected omega-3 FAs at 55 ˚C of temperature, 20 min of static extraction time, 60 min of dynamic extraction time, and 0:1 of co-solvent to feedstock mass ratio. Error bars show the standard error of the mean for duplicate samples, n=2.

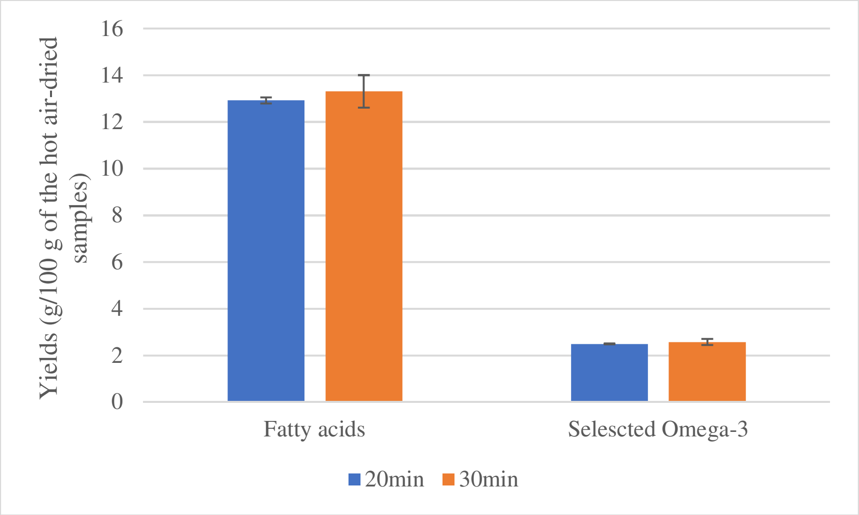

Figure S3. Effects of static extraction time (20 and 30 min) on yields of FAs and selected omega-3 FAs at 55 ˚C of temperature, 20 MPa of pressure, 60 min of dynamic extraction time, and 0:1 of co-solvent to feedstock mass ratio. Error bars show the standard error of the mean for duplicate samples, n=2.

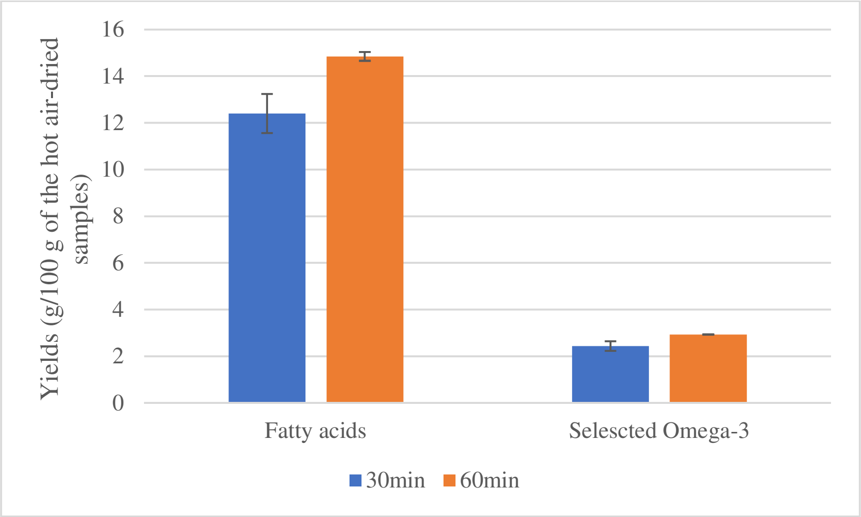

(a) Dynamic extraction time increased from 30 min to 60 min at 55 ˚C of temperature, 35 MPa of pressure, 20 min of static extraction time, and 0:1 of co-solvent to feedstock mass ratio.

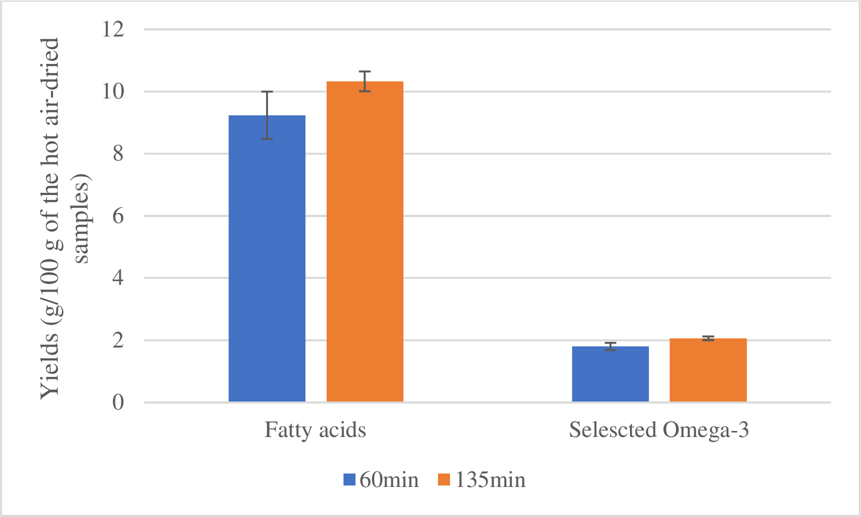

(b) Dynamic extraction time increased from 60 min to 135 min at 30 ˚C of temperature, 10 MPa of pressure, 0 min of static extraction time, and 0:0 of co-solvent to feedstock mass ratio.

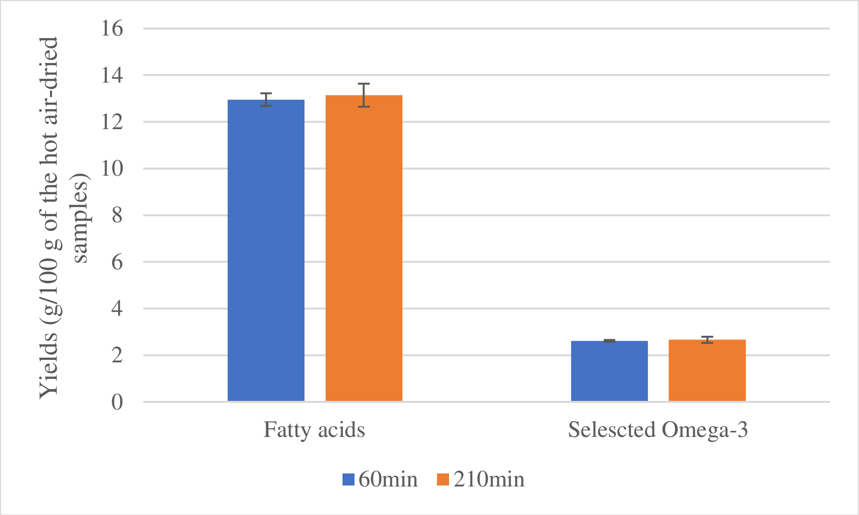

(c) Dynamic extraction time increased from 60 min to 210 min at 75 ˚C of temperature, 50 MPa of pressure, 20 min of static extraction time, and 0:0 of co-solvent to feedstock mass ratio.

Figure S4. Effects of dynamic extraction time on yields of FAs and selected omega-3 FAs. Error bars show Error bars show the standard error of the mean for duplicate samples, n=2.

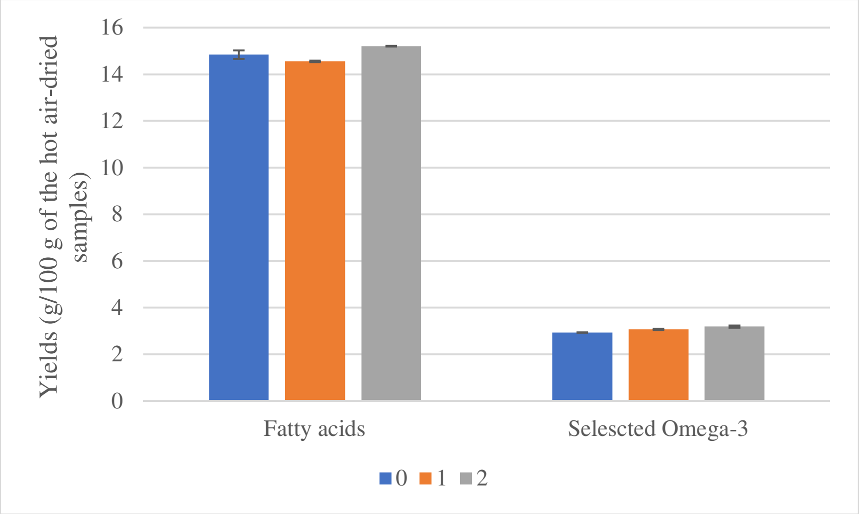

Figure S5. Effects of mass ratio of co-solvent to feedstock (0:1, 1:1, 2:1) on yields of FAs and selected omega-3 FAs at 55 ˚C of temperature, 35 MPa of pressure, 20 min of static extraction time, and 60 min of dynamic extraction time. Error bars show the standard error of the mean for duplicate samples, n=2.

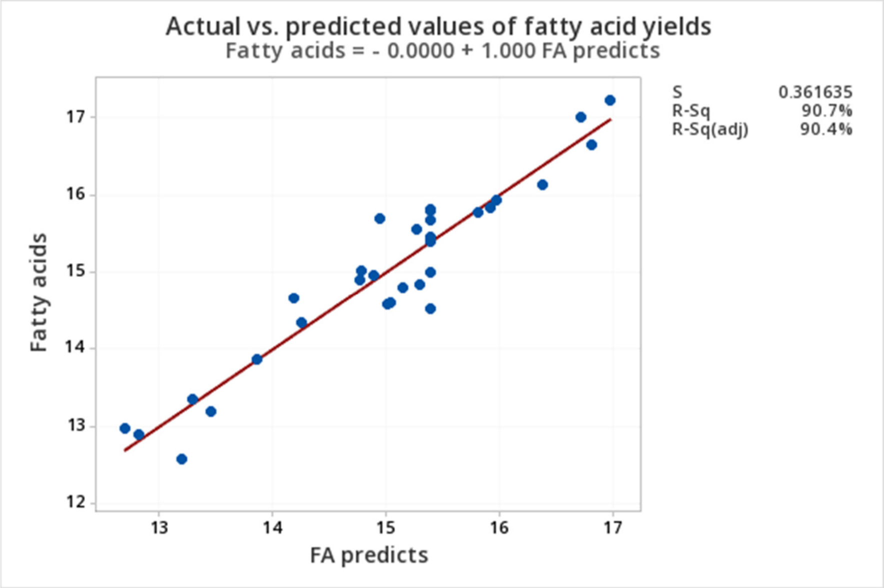

(a)

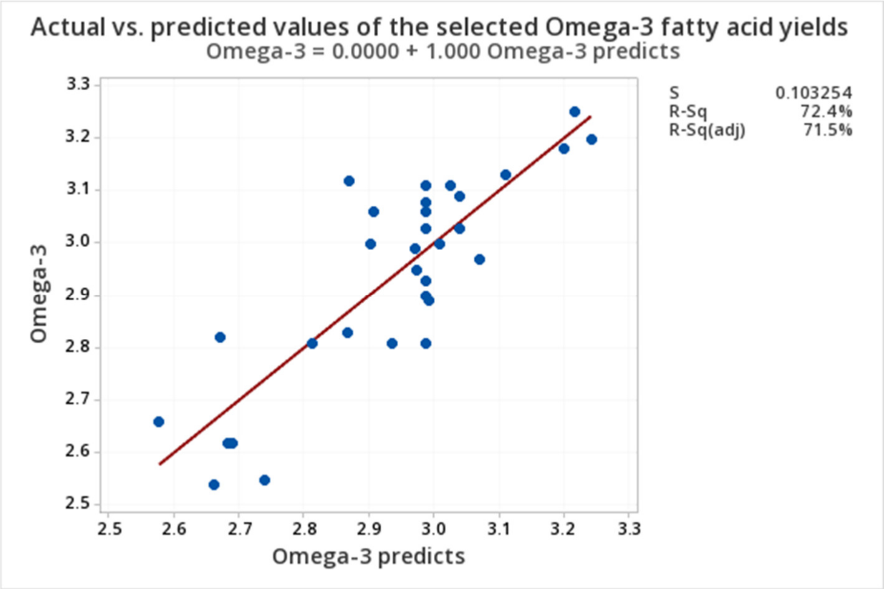

(b)

Figure S6. Actual vs. predicted values of yields of **(a)** FAs and **(b)** the selected omega-3 FAs.

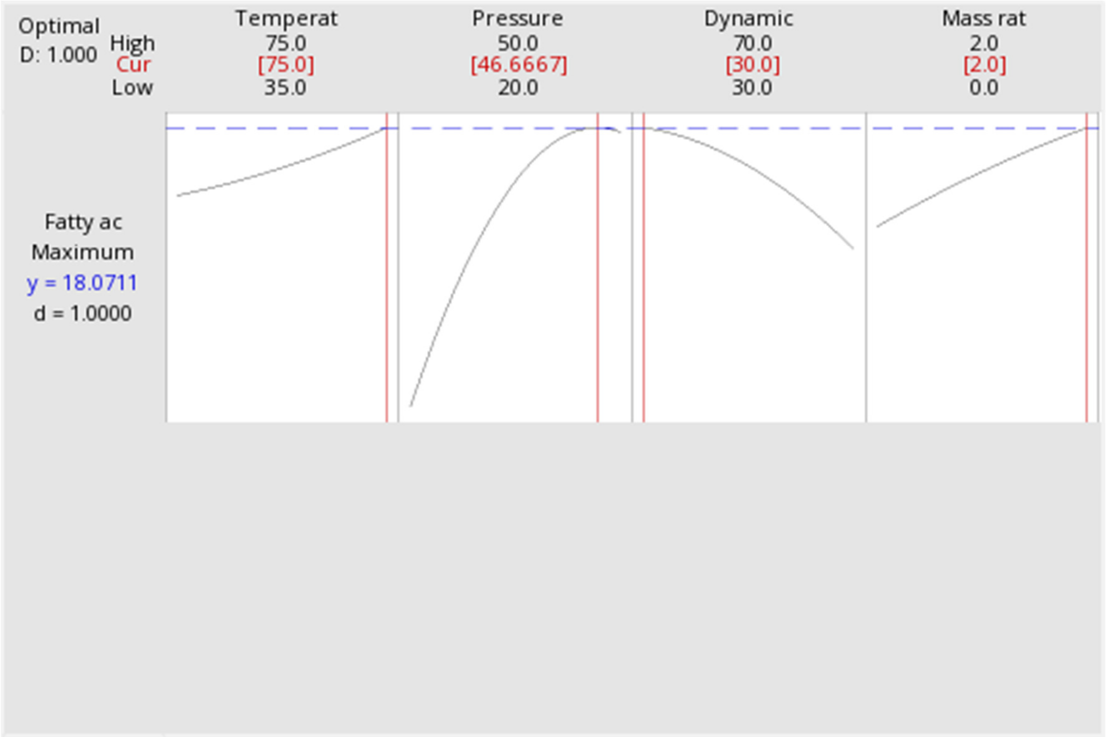
Optimal
D: 1.000
High
Cur
Low
Temperat
75.0
[75.0]
35.0
Pressure
50.0
[46.6667]
20.0
Dynamic
70.0
[30.0]
30.0
Mass rat
2.0
[2.0]
0.0
Fatty ac
Maximum
y = 18.0711
d = 1.0000

(a)

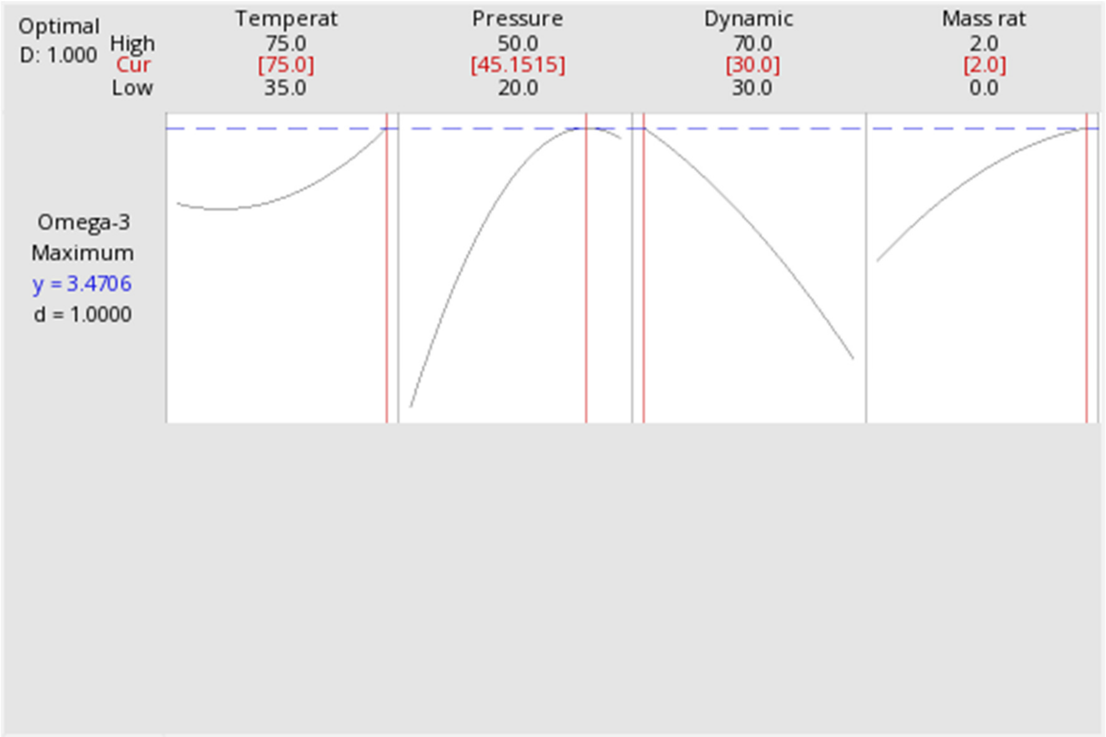
Optimal
D: 1.000
High
Cur
Low
Temperat
75.0
[75.0]
35.0
Pressure
50.0
[45.1515]
20.0
Dynamic
70.0
[30.0]
30.0
Mass rat
2.0
[2.0]
0.0
Omega-3
Maximum
y = 3.4706
d = 1.0000

(b)

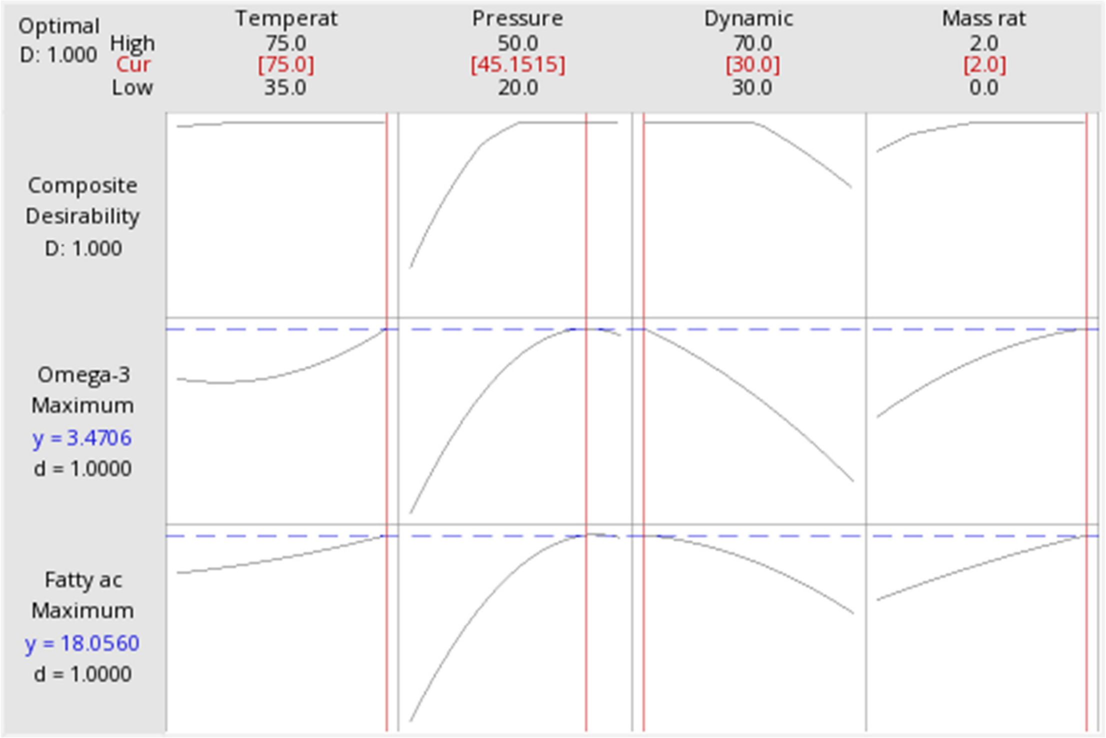

(c)

Figure S7. The optimum of $scCO_2$ extraction with co-solvent for **(a)** maximum FAs, **(b)** maximum the selected omega-3 FAs, and **(c)** maximum all responses.

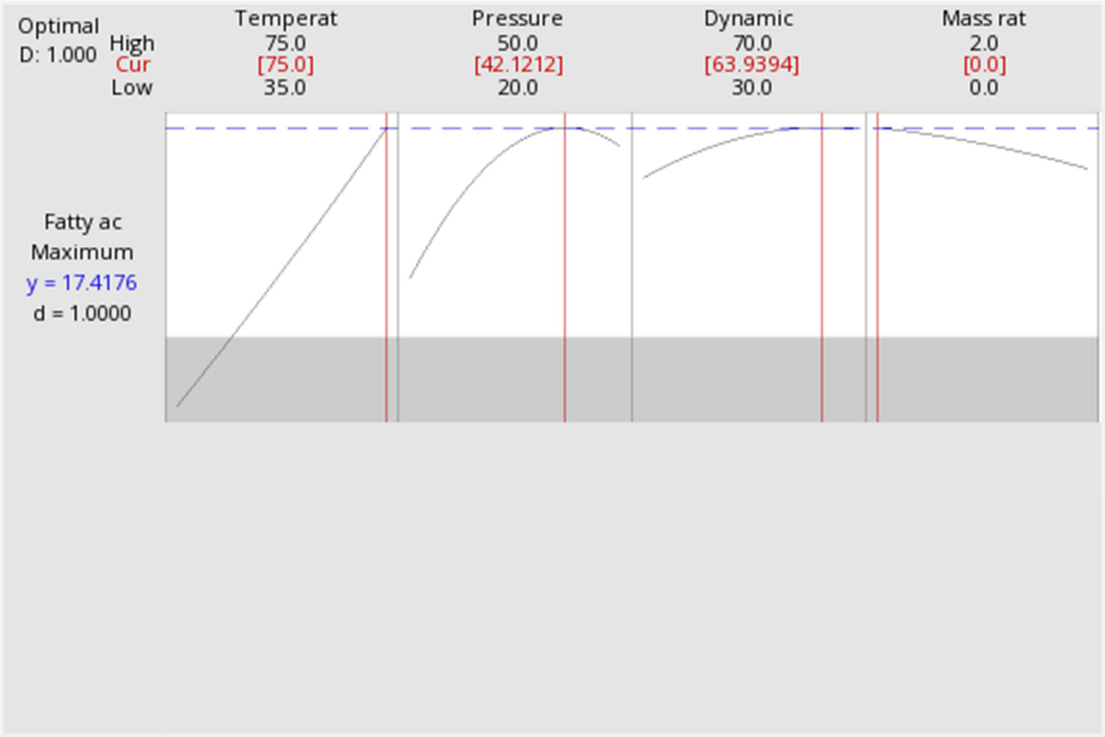
Optimal
D: 1.000
High
Cur
Low
Temperat
75.0
[75.0]
35.0
Pressure
50.0
[42.1212]
20.0
Dynamic
70.0
[63.9394]
30.0
Mass rat
2.0
[0.0]
0.0
Fatty ac
Maximum
y = 17.4176
d = 1.0000

(a)

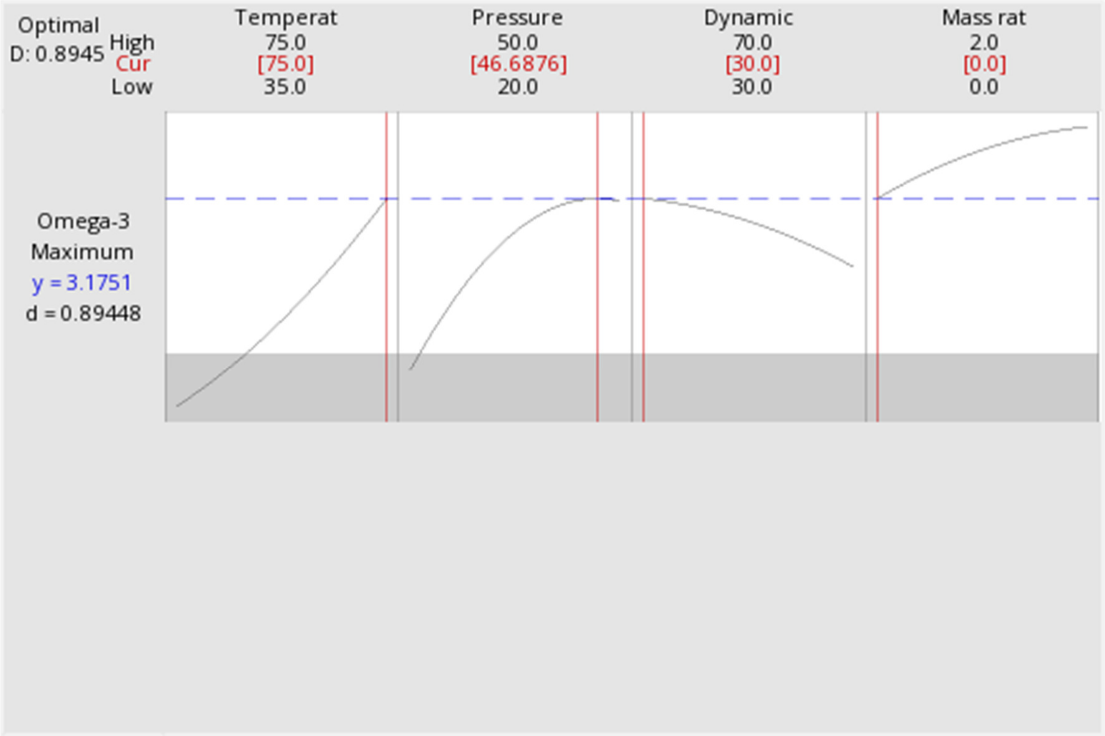
Optimal
D: 0.8945
High
Cur
Low
Temperat
75.0
[75.0]
35.0
Pressure
50.0
[46.6876]
20.0
Dynamic
70.0
[30.0]
30.0
Mass rat
2.0
[0.0]
0.0
Omega-3
Maximum
y = 3.1751
d = 0.89448

(b)

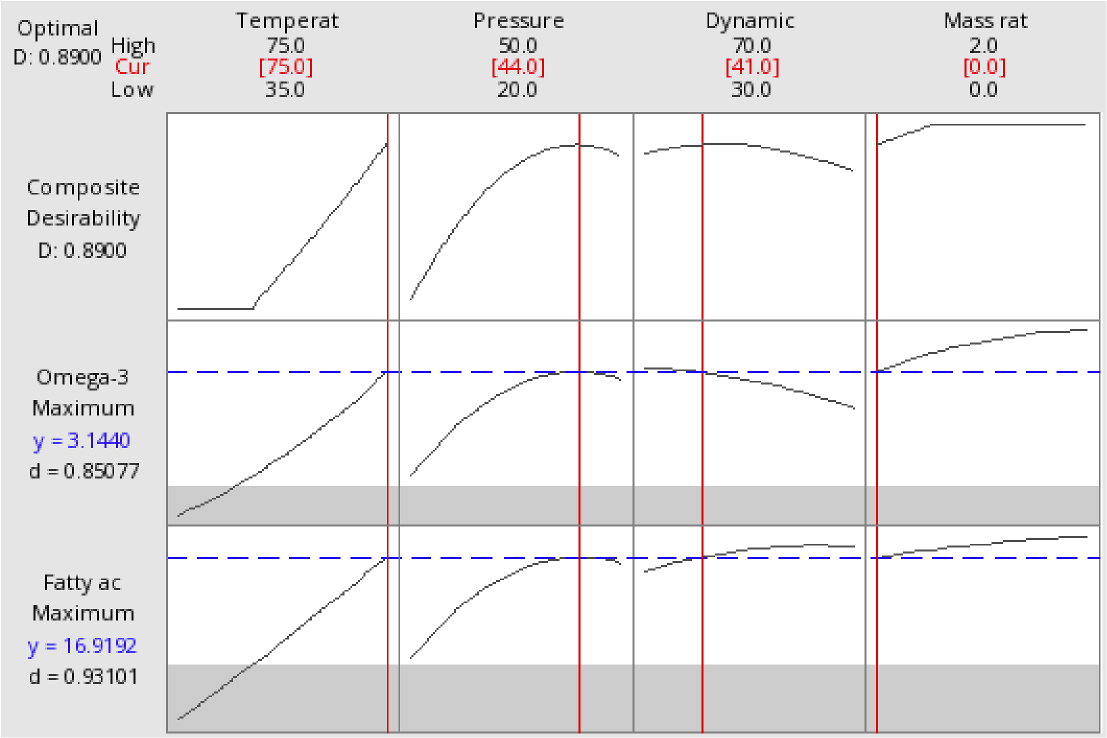

(c)

Figure S8. The optimum of $scCO_2$ extraction without co-solvent for **(a)** maximum FAs, **(b)** maximum the selected omega-3 FAs, and **(c)** maximum all responses.

Table S1. Central composite experimental design (inscribed) for $scCO_2$ extraction of omega-3 fatty acids (FAs) from sea cucumber viscera.

Run #	Temperature (˚C)	Pressure (MPa)	Dynamic Extraction Time (min)	Ratio of co-solvent to feedstock (w/w)
1	45 (-1)	27.5 (-1)	40 (-1)	0.5 (-1)
2	65 (+1)	27.5 (-1)	40 (-1)	0.5 (-1)
3	45 (-1)	42.5 (+1)	40 (-1)	0.5 (-1)
4	65 (+1)	42.5 (+1)	40 (-1)	0.5 (-1)
5	45 (-1)	27.5 (-1)	60 (+1)	0.5 (-1)
6	65 (+1)	27.5 (-1)	60 (+1)	0.5 (-1)
7	45 (-1)	42.5 (+1)	60 (+1)	0.5 (-1)
8	65 (+1)	42.5 (+1)	60(+1)	0.5 (-1)
9	45 (-1)	27.5 (-1)	40 (-1)	1.5 (+1)
10	65 (+1)	27.5 (-1)	40 (-1)	1.5 (+1)

11	45 (-1)	42.5 (+1)	40 (-1)	1.5 (+1)
12	65 (+1)	42.5 (+1)	40 (-1)	1.5 (+1)
13	45 (-1)	27.5 (-1)	60 (+1)	1.5 (+1)
14	65 (+1)	27.5 (-1)	60 (+1)	1.5 (+1)
15	45 (-1)	42.5 (+1)	60 (+1)	1.5 (+1)
16	65 (+1)	42.5 (+1)	60 (+1)	1.5 (+1)
17	35 (-2)	35 (0)	50 (0)	1 (0)
18	75 (+2)	35 (0)	50 (0)	1 (0)
19	55 (0)	20 (-2)	50 (0)	1 (0)
20	55 (0)	50 (+2)	50 (0)	1 (0)
21	55 (0)	35 (0)	30 (-2)	1 (0)
22	55 (0)	35 (0)	70 (+2)	1 (0)
23	55 (0)	35 (0)	50 (0)	0 (-2)
24	55 (0)	35 (0)	50 (0)	2 (+2)
25-31	55 (0)	35 (0)	50 (0)	1 (0)

Table S2. The CCI design of process variables with corresponding experimental responses (yields of the total FAs and the selected omega-3 FAs in g/100 g of samples on the dry weight basis), predicted values (predicted yields of the total FAs and the selected omega-3 FAs in g/100 g of samples on the dry weight basis), and recovery efficiencies (compared with the maximum FA contents obtained from *in-situ* transesterification of freeze-dried samples).

#	*Temp* (˚C)	*Press* (MPa)	*DET* (min)	*MRCSF* (w/w)	FAs yields			Selected omega-3 yields		
					Exp.	Predict	Recovery %	Exp.	Predict	Recovery %
1	45	27.5	40	0.5	12.99	12.70	58.61	2.66	2.58	64.37
2	65	27.5	40	0.5	14.35	14.26	64.73	2.81	2.82	67.98
3	45	42.5	40	0.5	13.37	13.31	60.34	2.55	2.74	61.75
4	65	42.5	40	0.5	15.79	15.82	71.21	3.03	3.04	73.31
5	45	27.5	60	0.5	12.59	13.21	56.81	2.62	2.69	63.26
6	65	27.5	60	0.5	14.91	14.78	67.26	2.84	2.87	68.53
7	45	42.5	60	0.5	13.21	13.46	59.60	2.62	2.69	63.33
8	65	42.5	60	0.5	15.94	15.98	71.90	2.81	2.94	68.01
9	45	27.5	40	1.5	14.82	15.15	66.88	2.99	2.97	72.26
10	65	27.5	40	1.5	15.57	15.27	70.24	3.09	3.04	74.67

11	45	42.5	40	1.5	15.84	15.92	71.47	3.13	3.11	75.70
12	65	42.5	40	1.5	17.24	16.99	77.78	3.20	3.24	77.29
13	45	27.5	60	1.5	14.98	14.90	67.58	3.00	3.01	72.50
14	65	27.5	60	1.5	14.59	15.02	65.83	3.11	3.03	75.29
15	45	42.5	60	1.5	14.85	15.31	66.99	2.89	2.99	69.88
16	65	42.5	60	1.5	16.14	16.38	72.83	2.97	3.07	71.92
17	35	35	50	1	14.68	14.19	66.21	3.00	2.90	72.44
18	75	35	50	1	16.65	16.82	75.12	3.25	3.22	78.51
19	55	20	50	1	12.91	12.83	58.24	2.54	2.662	61.41
20	55	50	50	1	15.03	14.80	67.81	3.12	2.87	75.41
21	55	35	30	1	14.61	15.04	65.90	2.95	2.97	71.41
22	55	35	70	1	15.70	14.95	70.85	3.06	2.91	73.88
23	55	35	50	0	13.89	13.87	62.68	2.82	2.67	68.11
24	55	35	50	2	17.02	16.73	76.78	3.18	3.20	76.79
25	55	35	50	1	15.69	15.39	70.78	3.06	2.99	74.07
26	55	35	50	1	15.46	15.39	69.73	3.03	2.99	73.27

27	55	35	50	1	15.81	15.39	71.34	2.93	2.99	70.80
28	55	35	50	1	15.83	15.39	71.41	3.11	2.99	75.17
29	55	35	50	1	15.02	15.39	67.76	2.81	2.99	67.99
30	55	35	50	1	14.54	15.39	65.58	2.90	2.99	70.01
31	55	35	50	1	15.41	15.39	69.54	3.08	2.99	74.52

Table S3. Analysis of variance (ANOVA) for response surface models of FA yields and the selected omega-3 FA yields (g/100 g of samples on a dry weight basis). Significant model items are in bold fonts.

	FA yields		Selected omega-3 FA yields	
	Coefficient	P-Value	Coefficient	P-Value
Model		0.000		0.019
Constant	-3.65		-0.44	
Linear		0.018		0.201
Temperature	-0.0021	0.975	-0.0022	0.908
Pressure	0.2166	**0.021**	0.0462	0.074
Dynamic extraction time	0.0881	0.191	0.0196	0.304

Co-solvent/feedstock mass ratio	3.57	**0.006**	0.525	0.124
Quadratic		0.008		0.274
Temperature*Temperature	0.00007	0.762	0.000045	0.495
Pressure*Pressure	-0.001759	**0.000**	-0.000247	**0.048**
Dynamic extraction time*Dynamic extraction time	-0.000249	0.291	-0.00003	0.655
Co-solvent/feedstock mass ratio*Co-solvent/feedstock mass ratio	-0.0245	0.791	-0.0131	0.622
2-way interaction		0.058		0.758
Temperature*Pressure	0.00079	0.069	0.000052	0.659
Temperature*Dynamic extraction time	0.000005	0.988	-0.000033	0.711
Temperature*Co-solvent/feedstock mass ratio	-0.01803	**0.009**	-0.00209	0.246
Pressure*Dynamic extraction time	-0.000298	0.473	-0.000131	0.274
Pressure*Co-solvent/feedstock mass ratio	0.00271	0.743	-0.00038	0.873
Dynamic extraction time*Co-solvent/feedstock mass ratio	-0.00959	0.134	-0.00084	0.634
Lack-of-Fit		0.466		0.216
R^2		90.69%		72.45%

Table S4. Optimal conditions of $scCO_2$ extraction of *C. frondosa* viscera (hot air-dried) for maximum FAs and the selected omega-3 FAs. The optimal conditions under different scenarios can be classified as co-solvent added and no co-solvent added.

Scenario	Optimal reaction conditions				95% predicted product yield interval[1] (g/100 g of samples on a dry weight basis)	
	Temp (˚C)	*Press* (MPa)	*DET* (min)	*MRCSF* (w/w)	FAs	Selected omega-3 FAs
Max FAs	75	47	30	2:1	(15.15, 20.99)	\
	75	42	64	0:1	(15.13, 19.71)	\
Max selected omega-3 FAs	75	45	30	2:1	\	(2.67, 4.27)
	75	47	30	0:1	\	(2.34, 4.01)
Max all responses	75	45	30	2:1	(15.24, 20.87)	(2.67, 4.27)
	75	44	41	0:1	(14.73, 19.11)	(2.52, 3.77)

[1]: a range containing a single response for the optimum, with 95% confidence.

Table S5. The FA profiles (weight percentage of total FAs) of the global extract of hot air-dried *C. frondosa* viscera obtained from the ultrasonic-assisted Bligh & Dyer method, the ultrasonic-assisted *in-situ* transesterification, and $scCO_2$ extraction (n=4, Mean±SD). Values in the same row with different letters are significantly different at $p < 0.05$.

Type	Isomer	Systematic name	Bligh & Dyer	*In-situ*	$ScCO_2$
Saturated	14:0	Myristic acid	1.74±0.01	1.87±0.02	2.00 ±0.04
	15:0	Pentadecylic acid	2.48±0.00	2.54±0.02	2.77±0.06
	16:0	Palmitic acid	1.44±0.00	1.43±0.01	1.52±0.05
	17:0	Heptadecanoic acid	0.27±0.01	0.30±0.01	0.30±0.03
	18:0	Stearic acid	2.99±0.02	2.80±0.03	2.63±0.01
	20:0	Arachidic acid	0.53±0.01	0.50±0.00	0.52±0.21
	21:0	Henicosanoic acid	0.20±0.00	0.19±0.00	0.16±0.01
	22:0	Behenic acid	0.62±0.01	0.53±0.02	0.48±0.02
Total			10.27±0.03[a]	10.17±0.09[a]	10.36±0.32[a]
Monounsaturated	16:1n-7	Palmitoleic acid	16.51±0.06	16.85±0.15	17.84±0.18
	17:1n-7	10-Heptadecenoic acid	0.44±0.00	0.45±0.01	0.48±0.00
	18:1n-9 cis	Oleic acid	1.30±0.01	1.31±0.01	1.29±0.01

	18:1n-7	Vaccenic acid	2.90±0.00	2.90±0.02	2.83±0.03
	20:1n-9	11-Eicosenoic acid	0.54±0.01	0.54±0.00	0.49±0.02
	22:1n-9	Erucic acid	0.50±0.02	0.53±0.03	0.44±0.02
	24:1n-9	Nervonic acid	1.86±0.04	1.73±0.02	1.57±0.08
Total			24.06±0.03[a]	24.30±0.21[a]	24.94±0.08[b]
Polyunsaturated	16:2n-4	Hexadecadienoic acid	0.93±0.04	1.01±0.03	1.03±0.06
	18:2n-6 trans	Linolelaidic acid	0.12±0.00	0.13±0.00	0.13±0.01
	18:2n-6 cis	Linoleic acid	0.28±0.00	0.28±0.00	0.27±0.00
	18:2n-4	11,14-Octadecadienoic acid	0.47±0.00	0.47±0.00	0.46±0.00
	20:2n-6	11,14-eicosadienoic acid	0.27±0.00	0.28±0.01	0.20±0.01
	22:2n-6	13,16-Docosadienoic acid	0.11±0.00	0.14±0.01	0.12±0.02
	16:3n-4	6,9,12-hexadecatrienoic acid	0.42±0.01	0.44±0.01	0.45±0.01
	18:3n-4	8,11,14-octadecatrienoic acid	0.10±0.01	0.10±0.00	0.10±0.01
	18:3n-3	α-Linolenic acid (ALA)	0.12±0.02	0.15±0.00	0.14±0.09
	16:4n-1	6,9,12,15-hexadecatetraenoic acid	1.22±0.01	1.26±0.01	1.28±0.01
	18:4n-3	Stearidonic acid	0.84±0.00	0.89±0.05	0.72±0.21

	20:4n-6	Arachidonic acid	0.57±0.00	0.60±0.01	0.46±0.08
	20:4n-3	Eicosatetraenoic acid (ETA)	0.28±0.01	0.30±0.01	0.21±0.02
	20:5n-3	Eicosapentaenoic acid (EPA)	22.49±0.04[a]	21.74±0.20[b]	19.83±0.48[c]
	21:5n-3	Heneicosapentaenoic acid (HPA)	0.39±0.03	0.37±0.00	0.63±0.01
	22:5n-3	Docosapentaenoic acid (DPA)	0.28±0.00	0.28±0.00	0.22±0.03
	22:6n-3	Docosahexaenoic acid (DHA)	1.23±0.02	0.88±0.01	0.88±0.04
Total of Omega-3			25.62±0.10[a]	24.59±0.25[b]	22.62±0.84[c]
Total of Omega-6			1.35±0.00[a]	1.43±0.01[a]	1.17±0.10[b]
Total			30.11±0.07[a]	29.30±0.30[a]	27.12±0.94[b]
	4,8,12-Me-13:0	4,8,12-Trimethyltridecanoic acid	2.48±0.01	2.58±0.02	2.82±0.07
	Me-14:0 isomer 1		0.47±0.00	0.58±0.00	0.58±0.04
	Me-14:0 isomer 2	Methyltridecanoic acid	0.41±0.02	0.41±0.00	0.46±0.01
Branched	Me-14:0 isomer 3		0.38±0.00	0.38±0.00	0.41±0.01
	i-16:0	14-methylpentadecanoic acid	4.35±0.01	4.40±0.03	4.76±0.09
	i-17:0	15-methylhexadecanoic acid	0.29±0.01	0.38±0.01	0.27±0.00
	ai-15:0	12-methyltetradecanoic acid	21.35±0.03[a]	22.06±0.20[a]	24.17±0.60[b]

	ai-17:0	14-methylhexadecanoic acid	0.87±0.02	0.82±0.02	0.96±0.06
Total			30.58±0.04[a]	31.61±0.28[b]	34.43±0.70[c]
Others[1]			4.98±0.03[a]	5.06±0.04[a]	3.15±0.13[b]

[1]*: Others refer to FA isomers presented in the sample GC chromatograph.*

Table S6. Ultrasonic-assisted *in-situ* transesterification of fresh, hot air-dried, and freeze-dried samples (n=4, Mean±SD), with contents of FAs and omega-3 FAs (g/100 g of samples on a dry weight basis) and moisture (wt.%). Values in the same column with different letters are significantly different at $p < 0.05$.

Pre-treatment	Moisture contents	FA contents	Selected omega-3 FA contents
Fresh samples	83.20±0.10[a]	5.09±0.69[a]	1.67±0.25[a]
Hot air dry	6.45±0.03[b]	17.43±0.20[b]	3.92±0.05[b]
Freeze dry	2.65±0.12[c]	22.17±0.73[c]	4.14±0.06[b]

Table S7. The FA profiles (weight percentage of the total FAs, n=4, Mean±SD) of the global extract of *C. frondosa* viscera obtained from $scCO_2$ extraction under the optimal condition. Viscera underwent different pre-treatments. Values in the same row with different letters are significantly different at $p < 0.05$.

Type	Isomer	Systematic name	Fresh	Hot air dry	Freeze dry	Fresh + EtOH	Hot air dry + EtOH
Saturated	14:0	Myristic acid	3.24±0.32	2.00±0.04	2.17±0.03	2.62±0.01	1.86±0.01
	15:0	Pentadecylic acid	1.32±0.15	2.77±0.06	2.35±0.04	1.54±0.01	2.60±0.02
	16:0	Palmitic acid	2.32±0.15	1.52±0.05	1.56±0.04	2.02±0.02	1.46±0.01
	17:0	Heptadecanoic acid	0.33±0.05	0.30±0.03	0.15±0.00	0.27±0.01	0.29±0.02
	18:0	Stearic acid	3.22±0.23	2.63±0.01	1.96±0.03	2.63±0.06	2.79±0.04
	20:0	Arachidic acid	0.50±0.04	0.52±0.21	0.34±0.01	0.49±0.01	0.47±0.01
	21:0	Henicosanoic acid	0.21±0.01	0.16±0.01	0.15±0.00	0.23±0.01	0.18±0.00
	22:0	Behenic acid	0.51±0.06	0.48±0.02	0.35±0.01	0.54±0.02	0.58±0.01
Total			11.65±0.47[a]	10.36±0.32[b]	8.99±0.11[c]	10.33±0.09[b]	10.23±0.02[b]
Monounsaturated	16:1n-7	Palmitoleic acid	19.19±0.70	17.84±0.18	20.78±0.41	19.67±0.07	17.13±0.05
	17:1	10-Heptadecenoic acid	0.37±0.04	0.48±0.00	0.45±0.01	0.39±0.00	0.46±0.01
	18:1n-9 cis	Oleic acid	2.16±0.02	1.29±0.01	1.52±0.05	2.10±0.02	1.33±0.00
	18:1n-7	Vaccenic acid	3.52±0.16	2.83±0.03	2.75±0.07	3.18±0.02	2.95±0.03

	20:1n-9	11-Eicosenoic acid	0.78±0.03	0.49±0.02	0.53±0.03	0.77±0.02	0.52±0.01
	22:1n-9	Erucic acid	0.67±0.07	0.44±0.02	0.41±0.02	0.67±0.02	0.53±0.01
	24:1n-9	Nervonic acid	1.80±0.26	1.57±0.08	1.26±0.11	1.79±0.08	1.92±0.06
Total			28.49±0.32[ac]	24.94±0.08[b]	27.69±0.69[c]	28.57±0.07[a]	24.84±0.02[b]
Polyunsaturated	16:2n-4	Hexadecadienoic acid	1.01±0.14	1.03±0.06	0.77±0.01	0.94±0.03	0.95±0.04
	18:2n-6 trans	Linolelaidic acid	0.11±0.01	0.13±0.01	0.07±0.01	0.12±0.02	0.17±0.03
	18:2n-6 cis	Linoleic acid	0.37±0.03	0.27±0.00	0.28±0.01	0.35±0.02	0.29±0.00
	18:2n-4	11,14-Octadecadienoic acid	0.39±0.02	0.46±0.00	0.36±0.03	0.37±0.00	0.46±0.00
	20:2n-6	11,14-eicosadienoic acid	0.26±0.02	0.20±0.01	0.17±0.00	0.24±0.01	0.22±0.01
	22:2n-6	13,16-Docosadienoic acid	0.16±0.01	0.12±0.02	0.16±0.03	0.19±0.01	0.12±0.00
	16:3n-4	6,9,12-hexadecatrienoic acid	0.65±0.02	0.45±0.01	0.43±0.02	0.56±0.01	0.43±0.01
	18:3n-4	8,11,14-octadecatrienoic acid	0.12±0.01	0.10±0.01	0.10±0.01	0.13±0.01	0.10±0.01
	18:3n-3	α-Linolenic acid (ALA)	0.19±0.01	0.14±0.09	0.11±0.06	0.18±0.02	0.22±0.00
	16:4n-1	6,9,12,15-hexadecatetraenoic acid	2.27±0.11	1.28±0.01	1.40±0.08	1.92±0.02	1.23±0.01
	18:4n-3	Stearidonic acid	1.12±0.03	0.72±0.21	0.69±0.04	0.97±0.01	0.84±0.00
	20:4n-6	Arachidonic acid	0.56±0.09	0.46±0.08	0.33±0.02	0.46±0.02	0.51±0.05
	20:4n-3	Eicosatetraenoic acid (ETA)	0.28±0.04	0.21±0.02	0.19±0.01	0.27±0.01	0.26±0.01
	20:5n-3	Eicosapentaenoic acid (EPA)	24.59±1.19[a]	19.83±0.48[bc]	18.62±1.46[c]	24.07±0.58[a]	21.29±0.21[b]

	21:5n-3	Heneicosapentaenoic acid (HPA)	0.79±0.10	0.63±0.01	0.59±0.12	0.82±0.01	0.65±0.03
	22:5n-3	Docosapentaenoic acid (DPA)	0.31±0.07	0.22±0.03	0.17±0.01	0.25±0.01	0.27±0.01
	22:6n-3	Docosahexaenoic acid (DHA)	1.25±0.11	0.88±0.04	0.85±0.07	1.28±0.02	0.98±0.01
Total of Omega-3			28.51±1.30[a]	22.62±0.84[bc]	21.20±1.60[b]	27.83±0.62[a]	24.50±0.22[c]
Total of Omega-6			1.46±0.10[a]	1.17±0.10[b]	1.01±0.04[c]	1.36±0.02[a]	1.31±0.04[ab]
Total			34.41±1.38[a]	27.12±0.94[bc]	25.26±1.73[b]	33.10±0.59[a]	28.99±0.16[c]
Branched	4,8,12-Me-13:0	4,8,12-Trimethyltridecanoic acid	1.98±0.15	2.82±0.07	2.42±0.04	1.94±0.03	2.63±0.03
	Me-14:0 isomer 1	Methyltridecanoic acid	0.40±0.03	0.59±0.04	0.61±0.05	0.46±0.01	0.59±0.01
	Me-14:0 isomer 2		0.30±0.03	0.46±0.01	0.52±0.01	0.37±0.01	0.43±0.01
	Me-14:0 isomer 3		0.16±0.03	0.41±0.01	0.34±0.01	0.20±0.00	0.38±0.00
	i-16:0	14-methylpentadecanoic acid	2.22±0.25	4.76±0.09	3.89±0.08	2.53±0.02	4.54±0.02
	i-17:0	15-methylhexadecanoic acid	0.17±0.02	0.27±0.00	0.27±0.00	0.19±0.00	0.26±0.00
	ai-15:0	12-methyltetradecanoic acid	14.78±1.23[a]	24.17±0.60[b]	26.76±0.49[c]	17.65±0.12[d]	22.53±0.17[b]
	ai-17:0	14-methylhexadecanoic acid	0.90±0.12	0.96±0.06	0.76±0.03	0.89±0.01	0.96±0.03
Total			20.90±1.67[a]	34.43±0.70[bd]	35.56±0.70[b]	24.23±0.15[c]	32.32±0.25[d]
Others[1]			4.55±0.58[a]	3.15±0.13[bc]	2.46±0.24[c]	3.77±0.50[ab]	3.62±0.07[b]

[1]: Others refer to FA isomers presented in the sample GC chromatograph.

References:

1. Jiao, G.; Kermanshahi pour, A. Extraction of Anthocyanins from Haskap Berry Pulp Using Supercritical Carbon Dioxide: Influence of Co-Solvent Composition and Pretreatment. *LWT* **2018**, *98*, 237–244, doi:10.1016/j.lwt.2018.08.042.
2. Maran, J.P.; Priya, B.; Manikandan, S. Modeling and Optimization of Supercritical Fluid Extraction of Anthocyanin and Phenolic Compounds from Syzygium Cumini Fruit Pulp. *Journal of Food Science and Technology* **2014**, *51*, 1938–1946, doi:10.1007/s13197-013-1237-y.
